# An Advanced High‐Performance Ultrafast Ammonium‐Ion Aqueous Battery Based on Dual‐Metal Redox Open Framework Molecular Magnet

**DOI:** 10.1002/advs.202514287

**Published:** 2026-01-15

**Authors:** Nilasha Maiti, Pramod Bhatt, Manoj K Sharma, Sher Singh Meena, Mayuresh D Mukadam, Soumen Samanta

**Affiliations:** ^1^ Solid State Physics Division Bhabha Atomic Research Centre Mumbai 400 085 India; ^2^ Homi Bhabha National Institute Anushaktinagar Mumbai 400 094 India; ^3^ Fuel Chemistry Division Bhabha Atomic Research Centre Mumbai 400 085 India; ^4^ Technical Physics Division Bhabha Atomic Research Centre Mumbai 400 085 India

**Keywords:** ammonium‐ion aqueous batteries, DFT, GITT, magnetization, Prussian blue analogs

## Abstract

We report a potassium manganese–iron hexacyanoferrate (KMnFeHCF) Prussian blue analog molecular magnet as a promising, low‐cost, environmentally friendly cathode for metal‐free ammonium‐ion aqueous batteries. KMnFeHCF crystallizes in a face‐centered cubic structure (*Fm3m*, lattice constant ≈ 10.19 Å) and exhibits a weak ferromagnetism, with Mössbauer spectroscopy confirming a mixed‐valence Fe⁺^3^/Fe⁺^2^ states. The material delivers a high specific capacity of ~145 mAh/g at 3 A/g, and ~130 mAh/g at 5 A/g along with excellent coulombic efficiency of 97%. Electrochemical performance is governed by reversible Fe²⁺/Fe³⁺ and Mn²⁺/Mn³⁺ redox transitions supported by the open‐framework tunnel‐like crystal structure which effectively accommodates structural distortions during ammoniation/de‐ammoniation. X‐ray photoelectron spectroscopy confirms mixed +2/+3 oxidation states for Fe and Mn. Density functional theory calculations show ammonium insertion induces tensile strain along Fe–C≡N–Mn linkages, expanding the lattice. The calculated migration barrier for NH_4_⁺ transport between 8c sites via the 24d site is 1.29 eV, reflecting favourable ion mobility. A full cell with a graphite anode achieves 71 mAh/g at 1.25 A/g and 51 mAh/g at 2.2 A/g, operating efficiently up to 1.8 V. It retains 50% capacity after 1850 cycles. Galvanostatic intermittent titration technique reveals a diffusion coefficient of 8.28 × 10^−8^ cm²/s, confirming fast transport kinetics.

## Graphical abstract

The Prussian Blue Analogue molecular magnet KMnFeHCF is demonstrated as a high‐performance cathode for ultra‐fast aqueous ammonium‐ion batteries. A full cell using KMnFeHCF and graphite delivers ~71 mAh g^−1^ at 1.25 A g^−1^ and ~51 mAh g^−1^ at 2.2 A g^−1^, retaining 50% capacity after 1850 cycles. Its scalability, cycling stability, and low cost offer strong application potential.

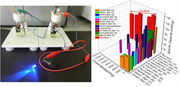

## 1 Introduction

In modern civilization, energy is a fundamental necessity that sustains our daily activities, and powers our economies. Therefore, the demand for energy is continuously increasing. Currently, fossil fuels such as coal, oil, and natural gas dominate as primary energy sources. However, these resources are finite, and their continued consumption leads to rapid depletion. Additionally, the extraction of fossil fuels incurs significant environmental costs, and burning them releases greenhouse gases that contribute to global warming and climate change. Hence, to meet energy demand together with net‐zero emissions, sustainable energy sources must be developed as alternatives to fossil fuels.^[^ ^1^, ^2^ ^]^ Although man‐made energy sources, including renewables like solar, wind, and hydropower, provide viable solutions, however, they are intermittent and reliant on weather conditions. Therefore, effective energy storage systems are essential to ensure reliability.^[^ ^3^ ^]^ In this regard, electrochemical energy storage systems like solid‐state batteries (SSBs) and supercapacitors are currently receiving extensive attention from researchers.^[^ ^3^, ^4^, ^5^, ^6^ ^]^ SSBs play a crucial role by storing energy through chemical reactions and converting it into electrical power for use when renewable energy sources are not available. Currently, lithium‐ion batteries (LIBs) dominate the market, serving applications from consumer electronics to electric vehicles.^[^ ^7^ ^]^ Despite their widespread use, LIBs face challenges such as the flammability and toxicity of organic electrolytes, as well as the rising costs of lithium due to its limited availability.^[^ ^8^, ^9^, ^10^ ^]^ Apart from the lithium, alternative metal ions like K⁺, Na⁺, Zn⁺^2^, Mg⁺^2^, Ca⁺^2^, and Al⁺^3^ are being explored as viable charge carriers.^[^ ^11^, ^12^, ^13^, ^14^, ^15^, ^16^, ^17^, ^18^, ^19^ ^]^ However, K⁺, Zn⁺^2^, and Al⁺^3^ have limitations such as corrosion of metal ions and phase changes during the ion insertion process of charging–discharging. To address these issues, metal‐free ions can be considered as charge carriers in combination with a liquid electrolyte. As a result, aqueous ion batteries (AIBs) are currently being explored.^[^ ^20^, ^21^, ^22^ ^]^ AIBs are based on the water electrolytes, making them nonflammable and reducing the risk of fire or explosion associated with organic electrolytes in SSBs. Moreover, AIBs offer certain advantages over SSBs. For example, water as an electrolyte is cheaper compared to the expensive and often toxic solvents used in SSBs, which lowers production costs. Furthermore, AIBs use nontoxic, environmentally friendly materials, making them more sustainable and easier to recycle. They also exhibit higher ionic conductivity, allowing for faster charge and discharge rates than solid electrolytes. Additionally, AIBs can be scaled for large‐scale energy storage applications, making them ideal for integrating renewable energy sources.

Currently, the majority of batteries utilize metal ions as charge carriers, with their performance governed by ion transport mechanisms. However, research on nonmetallic charge carriers, such as ammonium (NH_4_⁺) ion, and protons (H⁺), or hydronium (H_3_O⁺) is limited. Nonmetallic ions, especially NH_4_⁺, offer unique advantages like environmental significance and fast diffusion ability. Further, NH_4_⁺ ion is less corrosive and exhibits reduced hydrogen evolution. In addition, NH_4_⁺ ions are derived from abundant resources, have a low molar mass of 18 g mol^−1^, and exhibit rapid diffusion due to their small hydration radius, which enhances ionic conductivity in electrolytes. The hydrated NH_4_^+^ ion has a smaller radius of ≈3.31 Å than most of the hydrated metal ions. As a result, aqueous ammonium‐ion batteries (AAIBs) present a promising alternative to conventional metal‐ion‐based SSBs.^[^ ^23^, ^24^ ^]^ However, since AAIBs are in the initial stage of development, there are some challenges associated with them such as: i) the ionic radius of NH_4_^+^ ion is still quite large (≈1.54 Å), so the choice of electrode materials is limited. The electrode material must have an open tunnel‐like structure for NH_4_^+^‐ion insertion/extraction without compromising the structural stability. ii) The poor conductivity and sluggish kinetic behavior during the electrochemical reaction of NH_4_^+^ ions can lead to high polarization, resulting in poor rate performance of AAIBs. iii) The low output voltage and poor electrochemical performance due to the narrow voltage range and unstable interface of standard aqueous electrolytes. Therefore, finding a suitable electrode material with high specific capacity, cyclic stability, and rigid crystal structure for NH_4_^+^ ions has been the main bottleneck in developing AAIBs.

Prussian blue analogs (PBAs) or hexacyanoferrate compounds are very promising materials for AAIBs because of their low‐cost room‐temperature synthesis, structural flexibility, tunable electrochemical properties, and large open framework structure desired to achieve maximum performance of the batteries.^[^ ^22^, ^25^, ^26^ ^]^ The first study on AAIBs based on KM[Fe(CN)_6_] (M = Ni and Cu) PBAs was explored by Cui et al. in 2011.^[^ ^27^ ^]^ The reported PBAs can maintain a high electrochemical reaction potential in the half‐cell test of AAIBs.^[^ ^27^ ^]^ Subsequently, (NH_4_)_1.47_Ni[Fe(CN)_6_]_0.88_, PBA as the cathode of AAIBs has also been studied with specific capacity of 51 mAh g^−1^ at a current density of 300 mA g^−1^, and good cycling stability with a capacity retention of 74% after 2000 cycles.^[^ ^28^ ^]^ In recent times, a novel K–V–Fe PBA nanocube was synthesized using oxalic acid as a reducing agent in hydrothermal method.^[^ ^29^ ^]^ The compound shows a high specific capacity of 92.85 mAh g^−1^ at a current density of 2 A g^−1^, with outstanding cycling stability with a capacity retention of 91.44% (84.9 mAh g^−1^) after 2000 cycles at 2 A g^−1^ and good rate capability with a specific capacity of 46.2 mAh g^−1^ at 5 A g^−1^.^[^ ^29^ ^]^ The “common ion effect” also enhances its electrochemical performance as reported in iron hexacyanoferrate (FeHCF) PBAs for AAIBs.^[^ ^30^ ^]^ It is demonstrated that the initial capacity of FeHCF is about 80 mAh g^−1^ with a Coulombic efficiency of 97.8% and a retention rate of 96.3% over nearly 1000 cycles.^[^ ^30^ ^]^ Recently, hydrogen‐bond‐assisted ultrastable and fast aqueous NH_4_^+^‐ion storage properties of a cell fabricated using Cu‐based PBA as cathode and polyaniline as anode exhibited the feasibility of NH_4_^+^‐ion insertion/deinsertion for AIBs.^[^ ^31^ ^]^ The compound shows no capacity fading during ultralong cycles of 3000 times and high‐capacity retention of 93.6% at 50 C, confirming outstanding cycling and rate performance of Cu‐based PBA.^[^ ^31^ ^]^ A new design on AAIBs based on hydrogen‐bond‐anchored electrolyte, Mn‐based PBAs (MnHCF) cathode, and NaTi_2_(PO_4_)_3_@carbon anode has also been studied.^[^ ^32^ ^]^ The intermolecular hydrogen‐bond interaction is constructed between water and sulfolane molecules, leading to the decreased water activity and wide voltage window, resulting in stable cycling performance and high Coulombic efficiency.^[^ ^32^ ^]^ The organic NH_4_^+^‐ion battery using PBAs as cathode and 3,4,9,10‐perylenetetracarboxylic diimide (PTCDI) as the anode in a nonaqueous electrolyte has also been recently studied.^[^ ^33^ ^]^ The ball‐cutting of nanocube of sodium iron hexacyanoferrates PBAs, NaFe^+3^Fe^+2^(CN)_6_ was also investigated as a cathode material for AAIBs.^[^ ^34^ ^]^ The compound exhibits a high discharge capacity of 62 mAh g^−1^ at 0.25 A g^−1^ and 77.4% capacity retention at 2 A g^−1^. Furthermore, the compound shows cycling stability with no capacity loss over 50 000 cycles, due to the highly stable redox reaction of the high‐spin (HS) nitrogen‐coordinated Fe^+2^/Fe^+3^ couple.^[^ ^34^ ^]^ High‐performance AAIBs are developed by pairing Fe‐substituted manganese‐based PBAs as the cathode material with a highly concentrated NH_4_CF_3_SO_3_ electrolyte.^[^ ^35^ ^]^ The copper hexacyanoferrate PBA has been investigated for AAIBs, showing remarkable rate performance (60 mAh g^−1^ at 1 C, 59 mAh g^−1^ at 50 C) with no obvious capacity loss and negligible voltage polarization (≈0.1 V) from 1 to 50 C. The compound also shows capacity retentions of 91.5% and 86.5% are achieved over 17 000 and 20 000 cycles at 100 and 180 C, respectively.^[^ ^36^ ^]^ Recently, A novel entropy‐regulating strategy is proposed to simultaneously enhance the specific capacity and structural stability of PBAs. This is achieved by incorporating Cu, Ni, Co, Mn, and Fe into the 4b sites of the PBA framework, resulting in a multicomponent system referred to as CNCMF–PBAs. The synergistic effect of these randomly distributed metal ions generates a high density of redox‐active centers while also reinforcing the material's structural integrity.^[^ ^37^ ^]^ Although AAIBs offer safe, low‐cost, and eco‐friendly energy storage with fast NH_4_⁺ diffusion due to hydrogen bonding, their intercalation mechanisms especially at the atomic scale and structural stability are not yet fully understood. This is because research on AAIBs is still in its infancy compared to Li‐, Na‐, or Zn‐based systems.

Therefore, we investigate the detailed structural, magnetic, electronic, and electrochemical properties of potassium manganese–iron hexacyanoferrate (KMnFeHCF) material and successfully demonstrate its viability as a high‐performance cathode material for AAIBs. Density functional theory (DFT) calculations reveal a favorable NH_4_^+^‐ion activation energy and migration barrier, while Mn incorporation enhances charge storage and cycling stability by introducing additional redox‐active sites. With high specific capacity and exceptional cyclic stability, even at higher current densities, KMnFeHCF emerges as a promising low‐cost cathode for next‐generation metal‐free AAIBs.

## 2 Experimental Section

### 2.1 Synthesis of KMnFeHCF

The nanocrystalline single‐phase KMnFeHCF compound was prepared using the coprecipitation method. The precursor chemicals, iron(II) sulfate heptahydrate {Fe(SO_4_)·7H_2_O}, potassium hexacyanoferrate(III) {K_3_[Fe(CN)_6_]}, and manganese(II) sulfate monohydrate (MnSO_4_.H_2_O), were procured from Sigma‐Aldrich in reagent‐grade purity and used without further purification. Aqueous solutions of 0.15 m Fe(SO_4_)·7H_2_O, and 0.15 m MnSO_4_.H_2_O were each heated up to 50 °C for 10 min and then mixed together. The 0.1 m K_3_[Fe(CN)_6_] solution was then added to the mixed solution of Fe(SO_4_)·7H_2_O, and MnSO_4_.H_2_O under vigorous stirring to maintain uniform distribution of Fe and Mn in the solution. The resulting precipitate was thoroughly washed with doubly distilled water multiple times and subsequently air‐dried after filtration.

The film was prepared using the cyclic voltammetry (CV) method by varying the voltage from −0.2 to 1 V range. The anodic and cathodic peak current function of voltage was found to increase linearly with the electrodeposition up to 80 segments and increasing peak current confirmed the uniform layer‐by‐layer growth of the thin film. The potassium iron hexacynoferrate (KFeHCF) film was prepared by electrochemical reduction of ferric‐ferricyanide solution in acidic conditions as compared to neutral or basic environments, therefore, pH 2 solution was used in the sample preparation for greater structural stability. In neutral and alkaline conditions, ligand exchange occurred between the C≡N^−^ ligands and OH^−^ ions, leading to the degradation of the open framework like crystal structure.^[^ ^38^ ^]^ Electrochemical deposition of KMnFeHCF was carried out using three electrode cell system at ambient conditions. Indium‐tin‐oxide (ITO)‐coated glass substrate, platinum wire electrode, and Ag/AgCl (1 m KCl) electrode were used as the working, counter, and reference electrodes. The deposition bath contained solutions of 0.5 mm Fe_2_(SO_4_)_3_·7H_2_O, 0.5 mm MnSO_4_.H_2_O, and 1 mm K_3_[Fe(CN)_6_]. The electrochemical film deposition of KFeHCF and KMnFeHCF is shown in Figure S1 (Supporting Information).

### 2.2 Characterization of KMnFeHCF Compound

#### 2.2.1 Chemical Analysis

The chemical composition of the compound was analyzed using trace elemental analysis and combustion gas chromatography. The obtained elemental composition (wt%) was Mn: 9.1 (9.5), Fe: 19.4 (22.8), C: 16.1 (16.7), and N: 18.8 (19.6), where the values in bracket represented the theoretically expected values. The stoichiometry of the KMnFeHCF compound was found to be K_0.2_Mn_0.7_Fe_0.7_[Fe(CN)_6_].4.5H_2_O.

#### 2.2.2 Physical Characterization

The surface morphology and chemical composition of the compound were investigated using a field‐emission scanning electron microscope (FESEM) equipped with an energy dispersive X‐ray spectrometer. To minimize the charging effects during FESEM measurement, the powder sample was evenly sprinkled on the carbon tape mounted on an aluminum stub. An electron microscope (Model Carl Zeiss Auriga, with Gemini Column field emission gun) integrated with energy‐dispersive X‐ray spectroscopy (EDS) and electron backscatter diffraction detectors from Oxford Instruments was used for this purpose. Thermogravimetric analysis (TGA) was performed using a Linseis STA PT1600 instrument. The sample, along with an empty alumina crucible as a reference, was heated in an inert atmosphere up to 800 °C at a heating rate of 10 K min^−1^. The room temperature X‐ray diffraction (XRD) data for all compounds were recorded using a Rigaku diffractometer over an angular (2*θ*) range of 10°–65° using a Cu‐K_α_ (*λ* = 1.54 Å) radiation. Fullprof software,^[^ ^39^ ^]^ based on the Rietveld refinement method was used to simulate the *Fm*3*m* generated cubic structure. Fourier transform infrared (FTIR) spectroscopy data were recorded in reflectance mode using an attenuated total reflectance Alpha Platinum series spectrometer. The resolution of the FTIR instrument was 4 cm^−1^. X‐ray photoelectron spectroscopy (XPS) was performed to analyze the surface elemental composition and oxidation state of the atoms present in the compound. XPS measurement was carried out using a Mg‐Kα (1253.6 eV) X‐ray source and a DESA‐150 electron analyzer (M/s. Staib Instruments, Germany). The adventitious C‐1s peak, observed at 284.8 eV was used as the binding energy calibration reference. Temperature‐dependent DC magnetization measurements were performed down to 2 K under field‐cooled (FC) and zero‐field‐cooled (ZFC) conditions using a vibrating sample magnetometer from Cryogenic Ltd. Neutron depolarization measurement was conducted at the Dhruva reactor, BARC, India, using a neutron wavelength of 1.205 Å. The sample was initially cooled from room temperature to the lowest measurement temperature, 3.5 K under a 10 Oe field, and measurements were taken during the subsequent warming cycle. Mössbauer spectroscopy in transmission geometry, operating in constant acceleration mode, was carried out at room temperature to analyze the ionic state and local environment of Fe ions in the compound. A 50 mCi Co‐57 source in a Rh matrix was used for the measurements. High‐resolution transmission electron microscopy (HRTEM) and selected area electron diffraction (SAED) images were captured using a Field Emission Gun‐Transmission Electron Microscope at 300 kV electron source.

#### 2.2.3 Electrochemical Characterization

Electrochemical experiments were conducted using a CHI 608 electrochemical workstation in a two electrode and three‐electrode setup. The working electrode was composed of the active material KMnFeHCF, while a Ag/AgCl (1 m KCl) electrode served as the reference, and a platinum electrode functioned as the counter electrode for three electrode setups. Two‐electrode cell, comprised of KMnFeHCF cathode and a graphite anode. Aqueous 0.1 m (NH_4_)_2_SO_4_ solution was used as the electrolyte. The (NH_4_)_2_SO_4_ electrolyte was chosen because it had high solubility, good ionic conductivity, and was compatible with PBA electrodes. A pH of ≈6.2 was selected since alkaline conditions caused electrode degradation through M─CN bond hydrolysis, while acidic conditions led to hydrogen evolution. Therefore, a near‐neutral pH helped to prevent side reactions and potential gas evolution. As a result, no visible gas release or electrode damage was observed even after long‐term cycling. To prepare the working electrode, the synthesized active material (70 wt%) was blended with carbon black (15 wt%) and polyvinylidene fluoride (15 wt%) in *N*‐methyl‐2‐pyrrolidone, forming a homogeneous slurry. This slurry was then coated onto an activated carbon cloth substrate and dried for 4 h using vacuum oven. The final electrode had an approximate mass of ≈1 mg and dimensions of 1 × 1 cm^2^, optimized after several experimental trials. It was observed that excessive material loading led to erosion or peeling from the electrode substrate. Although the loading amount was relatively low, it provided the best performance on the carbon cloth substrate. The electrochemical performance of KMnFeHCF as a cathode material for AAIBs was investigated using galvanostatic charge–discharge (GCD) measurements. Galvanostatic intermittent titration technique (GITT) measurement was carried out using the Neware battery testing system BTS4000.

### 2.3 Computational Methods: Density Functional Theory Calculation

All DFT calculations were performed using the Vienna ab initio simulation package (VASP 5.4.4) with a plane‐wave basis set and the projected augmented wave method to account for the interactions between core electrons and valence electrons. The generalized gradient approximation of the Perdew–Burke–Ernzerhof functional was utilized as the exchange correlation functional. The cutoff energy of the planewave basis was set at 500 eV. In the process of structural optimization, the Hellmann–Feynman forces were converged to 0.05 eV Å^−1^, and the energy convergence criterion for each atom was set at 10^−5^ eV. The Monkhorst–Pack method was used to sample the Brillouin‐zone with a *k*‐point mesh of 2 × 2 × 2. Then, DFT calculations were performed to confirm the low‐energy model configurations of the PBAs when NH_4_^+^ ions were inserted.

## 3 Results and Discussion

### 3.1 XRD, FESEM, FTIR, and TGA Study

**Figure 1:**
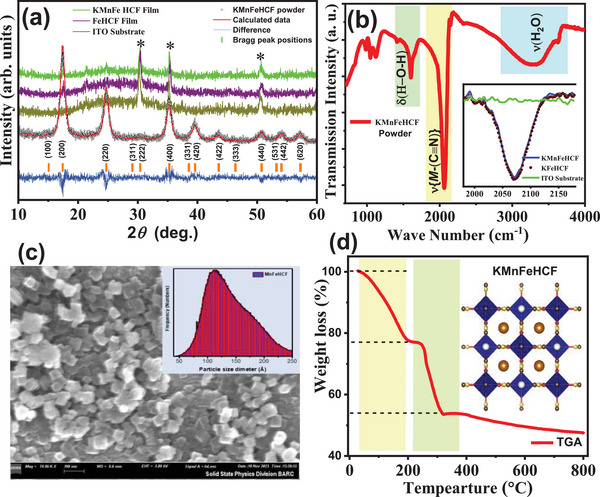
a) Room temperature powder XRD pattern of KMnFeHCF compound fitted using Rietveld refinement along with XRD patterns of KMnFeHCF and KFeHCF films deposited on ITO substrate. b) The peaks marked with (*) are the Bragg Peaks originated from the bare ITO substrate. The FTIR spectra of KMnFeHCF and KFeHCF compounds. c) FESEM image of the KMnFeHCF compound along with their size distribution in the inset. d) TGA data of KMnFeHCF compound. A schematic cubic crystal structure (inset of d) of the PBA compound showing metal ion is in octahedral arrangement. The water molecules or alkali metal ions are located at the intertrial space.

**Figure** **1** presents structural and morphological studies of the KMnFeHCF compound. Figure 1a shows room temperature XRD patterns of the KMnFeHCF compound in powder as well as in thin film form. The Rietveld analysis of the powder sample confirms a single nanocrystalline phase of the compound with the *Fm*3*m* space group. The lattice constant is found to be ≈10.19 (7) Å for the compound. The crystal structure analysis of KMnFeHCF compound reveals that the Fe^+2^/Mn^+2^ atoms located at the 4a (0, 0, 0) crystallographic position, whereas, Fe^+3^ atoms occupy the 4b (½, ½, ½) crystallographic position. The Rietveld refined structural parameters for the KMnFeHCF compound is presented in **Table** **1**. The XRD patterns of thin films of KMnFeHCF and KFeHCF compounds along with bare ITO substrate are also shown in Figure 1a. Both KFeHCF and KMnFeHCF films exhibit low intensity Bragg peak (200), corresponding to bulk compound, confirming the formation of single phase, crystalline face cantered cubic (fcc) structure. The crystallite size (*t*) of the compound was estimated using the Scherrer formula, *t* = 0.9*λ*/*β*cos*θ*, where *λ* represents the X‐ray wavelength, *β* is the line broadening at half maximum intensity measured in radians, and *θ* is the Bragg angle. The most intense Bragg peak (200) has been used for line broadening. The crystallite size is found to be ≈100 Å.

**Table 1:** Rietveld refined structural parameters for the KMnFeHCF compound. The fractional coordinates are denoted as *x’*, *y’*, and *z’*.

	Atom	Wyckoff site	*x’*	*y’*	*z’*	Occupancy
Space Group	Fe	4a	0	0	0	0.12
	Mn	4a	0	0	0	0.35
*Fm*3*m*	Fe	4b	0.5	0.5	0.5	0.31
Lattice constants	C	24e	0.32 (1)	0	0	0.51
*a* = *b* = *c* = 10.19 (7)	N	24e	0.19 (5)	0	0	0.27
	O_1_	24e	0.18 (7)	0	0	0.12
	O_2_	32f	0.256 (4)	0. 256 (4) (4) (5)	0. 256 (4)	0.18
	K	8c	0.25	0.25	0.25	0.02

Figure 1b shows FTIR study of the KFeHCF and KMnFeHCF compounds along with the bare ITO substrate. The presence of stretching frequencies of ν(C≡N) in the spectral range of 1900–2200 cm^−1^ confirms the formation of cyanoferrate compounds. The free C≡N ion shows stretching frequency at ≈2060 cm^−1^ in aqueous solution, however, when it bonds with metal ions, it shifts to a higher wavenumber side. In addition, oxidation state, electronegativity, and the nature of the metal ions coordinated to the cyanide ion and their coordination numbers also change the stretching frequency of the C≡N ion. Both films show strong absorption band at 2070 cm^−1^ attributed to the C≡N functional group.^[^ ^40^, ^41^ ^]^ Apart from the main intense peak, there is a low‐intensity broad stretching frequency peak at 2078 cm^−1^ for the KMnFeHCF film which is attributed to the characteristic peak of the ─C≡N group within the Fe^+3^─C≡N─Mn^+2^.^[^ ^42^, ^43^ ^]^ Figure 1c shows the FESEM images of KMnFeHCF film and particle size distribution. The FESEM images show uniformly distributed nanocube‐like particles. The particle size distribution, shown in the inset of Figure 1c, is derived from the histogram. The average particle size is found to be ≈125 Å for KMnFeHCF. The EDS elemental mapping of the KMnFeHCF compound is presented in Figure S2 (Supporting Information).

The TGA curve of KMnFeHCF compound is shown in Figure 1d. An initial weight loss of ≈23% is observed up to around 200 °C, attributed to the release of both uncoordinated and coordinated water molecules, located at the interstitial (8c and 32f) and coordinated (24e) sites within the PBA crystal structure. This corresponds to the removal of nearly ≈5 water molecules per formula unit. As the temperature increases from 247 to 322 °C, an additional weight loss of about 22% occurs. Beyond 322 °C, the compound begins to decompose, resulting in a total weight loss exceeding 50%. A schematic diagram of the cubic structure of KMnFeHCF is shown in Figure 1d in which Fe^+2^/Mn^+2^ ions are coordinated by six nitrogen atoms and Fe^+3^ ions are coordinated by six C atoms as shown in an octahedral geometry of HS and low‐spin (LS) sites, respectively. Water molecules at an interstitial crystallographic site (8c and 32f) and coordinated sites (24e) are known as noncoordinated water and coordinated water, respectively. By introducing alkali ions such as K^+^, Na^+^, Li^+^, and NH_4_^+^ at the interstitial positions, the stoichiometry of the compound can be varied resulting in changes in the number of vacancies and water molecules in the crystal structure.^[^ ^44^ ^]^ For example, A_3_[B(CN)_6_]_2_·*z*H_2_O stoichiometric PBA compound (where A and B are transition metal ions), ≈33%, B(C≡N)_6_ vacancies are present in the compound resulting both coordinated and noncoordinated water molecules. However, with the introduction of alkali ions (X) at the interstitial sites, the stoichiometry of the compound changes to XA[B(CN)_6_]·*z*H_2_O, resulting in the absence of vacancies and coordinated water.

### 3.2 DC Magnetization, Mössbauer, and Neutron Depolarization Study

**Figure 2:**
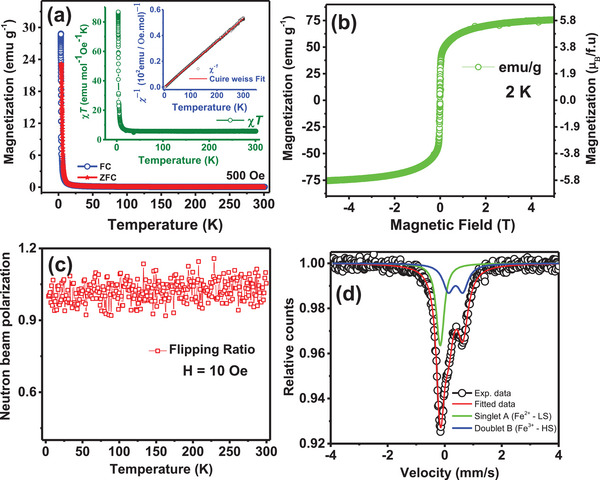
a) FC and ZFC magnetization (*M*) as a function of temperature (*T*) for the KMnFeHCF compound at 500 Oe field. The *χT* versus temperature (*T*) and the inverse susceptibility (*χ* ^−1^) versus *T* curves for the compound are shown in the inset of (a). The red line shows the Curie–Weiss law fitting of the inverse susceptibility (*χ* ^−1^) versus *T* curve. b) Magnetization as a function of magnetic field at 2 K for the KMnFeHCF compound. c) Temperature‐dependent neutron depolarization data of the KMnFeHCF compound recorded at 10 Oe. d) Room temperature Mössbauer spectrum of KMnFeHCF powder sample. The recorded experimental data (open circles) of powder sample are fitted with singlet and doublet shown by the thick green and blue lines, respectively.

The magnetic properties of KMnFeHCF are investigated using FC and ZFC magnetization, Mössbauer spectroscopy, and neutron depolarization studies. The FC and ZFC magnetization curves (**Figure** **2**a) show a weak ferromagnetic‐like transition near 5 K. The product of susceptibility (*χ*) and temperature (*T*) (*χT*) versus *T* curve (inset of Figure 2a) gives a room‐temperature value of 5.7 emu mol^−1^ Oe^−1^ K^−1^, consistent with HS Fe⁺^3^ in an octahedral environment and similar to that of Prussian blue (PB) compound, Fe_4_[Fe(CN_)6_]_3_.*z*H_2_O. The PB compound exhibits a ferromagnetic transition at ≈5.6 K via a double‐exchange mechanism between Fe⁺^2^ and Fe⁺^3^. In KMnFeHCF, the larger lattice parameter (≈10.19 Å) weakens electronic exchange through cyanide bridges, leading to a lower ordering temperature. Additionally, its nanocrystalline forms, such as nanocubes, nanospheres, nanorods, may also exhibit spin‐glass behavior with decreasing particle size.^[^ ^45^, ^46^, ^47^ ^]^ The manganese iron‐based compound of stoichiometry, Mn_3_[Fe(CN)_6_]_2_·*z*H_2_O exhibits antiferromagnetic transition below 9 K. The inverse of the susceptibility (*χ* ^−1^) as a function of temperature (*T*), fitted using the Curie–Weiss law, gives a Weiss temperature of 2 K, indicating a weak ferromagnetic exchange interaction. The experimental effective magnetic moment (*µ*_eff_ ≈ 4.17 µB f.u.^−1^) closely matches the theoretical spin‐only value of 4.5 µB f.u.^−1^ Figure 2b shows the magnetization (*M*) as a function of the applied magnetic field (*H*) measured up to ±5 Tesla. The shape of *M*–*H* loop at 2 K shows no coercivity. The saturation magnetization is found to be 5.8 µB f.u.^−1^ at 2 K. Figure 2c shows the neutron depolarization data using polarized neutron scattering for the KMnFeHCF compound. The compound does not show any depolarization of the polarized neutron beam down to ≈4 K confirms the absence of long range ferromagnetic domain in the compound.^[^ ^48^ ^]^

Figure 2d shows a Fe Mössbauer spectrum of KMnFeHCF bulk powder recorded at ±4 mm s^−1^, fitted with a singlet and a symmetric doublet. The fitted hyperfine parameters, including the isomer shift (*δ*), line width (*Γ*), and quadrupole splitting (*∆*), are presented in the Table S1 (Supporting Information). The parameters for the singlet and doublet indicate that Fe ions have two different crystallographic sites or environments in cubic symmetry. The value of *δ* of the singlet confirms that the Fe ions are in the LS Fe^+2^ (𝑡_2𝑔_^6^𝑒_𝑔_^0^, Fe^LS^, *S* = 0) state in a cubic octahedral molecular symmetry, showing no quadrupole splitting.^[^ ^49^, ^50^ ^]^ In contrast, the value of *δ* for doublet confirms that Fe ions are in HS Fe^+3^ (𝑡_2𝑔_^3^𝑒_𝑔_^2^, Fe^HS^, *S* = 5/2) state.

**Figure 3:**
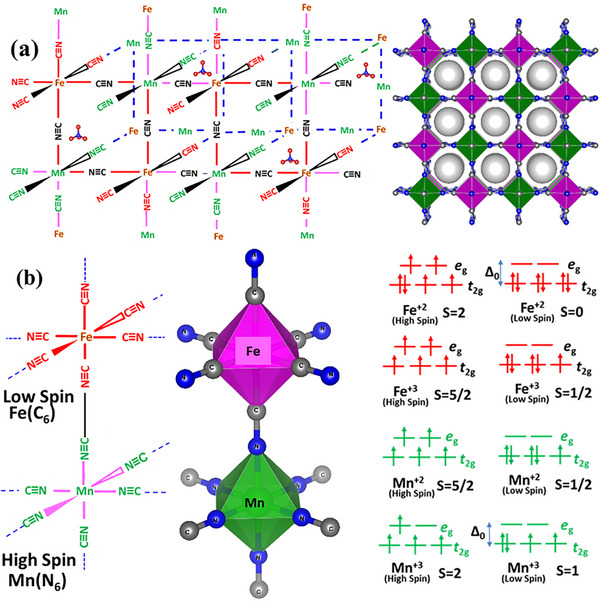
a) The schematic diagram representing crystal lattice structure of KMnFeHCF compound. b) The electronic configuration of HS and LS state of Fe and Mn atoms.

The two dimentional (2D) and three dimentional (3D) arrangements of Mn and Fe atoms connected via C≡N ligands in the crystal structure of KMnFeHCF are schematically illustrated in **Figure** **3**a. The 2D arrangement clearly shows that each Fe atom is coordinated with six carbon atoms, while each Mn atom is coordinated with six nitrogen atoms, forming octahedral geometries around both metal centers. This variation is attributed to the strong field strength of the cyanide ligands, which induce a splitting of the d orbitals into t_2g_ and e_g_ orbitals, leading to a LS and HS configuration in the compound. Figure 3b presents the spin‐state configuration, highlighting the LS state of Fe atoms and the HS state of Mn atoms along with their corresponding electronic configurations. The fitted room temperature Mössbauer spectra of the compounds such as Fe(SO_4_), Fe_2_(SO_4_)_3_, and K_3_[Fe(CN)_6_] along with KFeHCF are presented in Figure S3 (Supporting Information).

The electronic configuration of transition‐metal ions strongly influences redox kinetics and ion diffusion. For instance, Mn⁺^3^ in a HS d⁴ state (t_2g_^3^e_g_^1^, *S* = 2) undergoes Jahn–Teller distortion, elongating MnN_6_ octahedra and creating a polarized lattice. This increases the activation energy for electron transfer, slowing redox kinetics at high discharge rates. By contrast, the Fe⁺^3^/Fe⁺^2^ redox couple, particularly in the LS state, involves t_2g_ orbitals with minimal geometric distortion, thereby facilitating faster and more reversible electron transfer. Thus, distinguishing HS versus LS states directly informs our understanding of the electrochemical behavior of KMnFeHCF.

### 3.3 Electrochemical Study

**Figure 4:**
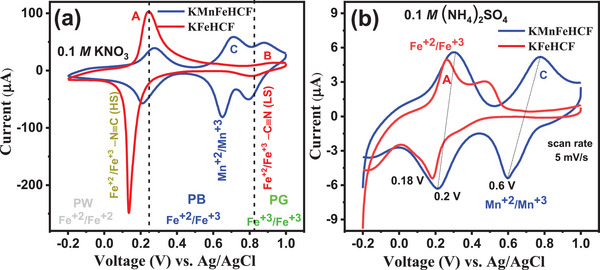
a) Cyclic voltammetry (CV) curves of KMnFeHCF and KFeHCF compounds in 0.1 m KNO_3_ electrolyte at a scan rate of 5 mV s^−1^. b) The dashed line shows the boundary for the evolution of Prussian white (PW), PB, and Prussian green (PG) with increasing voltage. CV curves of KMnFeHCF and KFeHCF compounds in 0.1 m (NH_4_)_2_SO_4_ electrolyte at a scan rate of 5 mV s^−1^.

**Figure** **4**a shows CV curves of KFeHCF and KMnFeHCF compounds, recorded at a scan rate of 5 mV s^−1^ in 0.1 m KNO_3_ electrolyte solution. The CV curve of KFeHCF shows two pairs of redox peaks mainly at ≈0.24/0.15 V (marked by A) and 0.8/0.9 V (marked by B), respectively. These peaks originate due to the redox‐active Fe^+2^/Fe^+3^ couples of HS N‐coordinated Fe^+3^ {Fe^+3^─N≡C}, and LS C‐coordinated Fe^+2^ {Fe^+2^─C≡N}, respectively.^[^ ^51^ ^]^ Whereas, additional peaks, observed at 0.7/0.65 V (marked by C) for KMnFeHCF are correspond to Mn^+2^/Mn^+3^ redox couples of LS C‐coordinated Mn^+2^ (Mn^+2^─C≡N). The evolution of PB (0.24–0.82 V), Prussian green (PG) (0.82–1 V), and Prussian white (PW) (−0.2–0.24 V) as a function of voltage is marked by the dotted line. In order to study the NH_4_^+^ insertion/extraction in KFeHCF and KMnFeHCF compounds for the AIBs, CV and GCD measurements are carried out in 0.1 m (NH_4_)_2_SO_4_ electrolyte. The CV curves recorded at a scan rate of 5 mV s^−1^ for KFeHCF and KMnFeHCF compounds in 0.1 m NH_4_(SO_4_)_2_ electrolyte solution, as shown in Figure 4b. The Fe and Mn redox peaks for both compounds are modified in the presence of NH_4_^+^ ions.

The CV of KFeHCF shows two oxidation redox peaks at 0.26 and 0.48 V and one reduction peak at 0.18 V which are attributed to Fe^+3^/Fe^+2^ couple of HS N‐coordinated Fe^+3^.^[^ ^34^ ^]^ It is observed that the LS C‐coordinated Fe^+3^ is redox inactive in the NH_4_^+^ electrolytes. However, the CV curves for KMnFeHCF show well‐defined two pair of redox peak due to Fe and Mn. The peak observed at 0.3/0.2 V is associated to Fe^+3^/Fe^+2^ couple of HS N‐coordinated Fe^+3^ and 0.7/0.6 V are associated to Mn^+3^/Mn^+2^ couple of LS C‐coordinated Mn^+2^. Furthermore, the presence of the Mn redox peak increases the area under the CV curve, confirming the superior electrochemical performance of KMnFeHCF compound.

**Figure 5:**
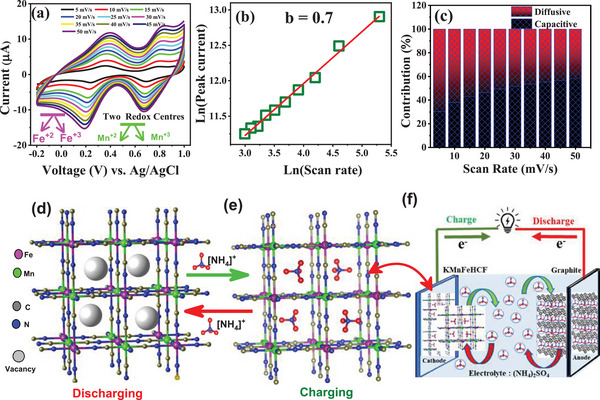
a) CV curves of KMnFeHCF compound in 0.1 m (NH_4_)_2_SO_4_ electrolyte at different scan rates. b) Linear fitting (red line) of the peak current (*I*_p_) versus scan rate (in mV s^−1^). c) The diffusion and capacitive controlled contribution to the charge storage kinetics. Crystal structure of KMnFeHCF compound during d) discharging (presence of vacancies) and e) charging state (presence of NH_4_^+^ ion). f) A schematic diagram of aqueous NH_4_^+^‐ion battery based on KMnFeHCF as cathode material and graphite as anode material is presented.

**Figure** **5**a shows the CV curves of KMnFeHCF compound at different scan rates (5, 10, 15, 20, 25, up to 50 mV s^−1^) in 0.1m (NH_4_)_2_SO_4_ electrolyte. The cyclic stability of the compound was initially evaluated over 15 cycles, confirming that the compound is both electrochemically active and stable (Figure S4, Supporting Information). At lower scan rates, the CV curves exhibit low‐intensity redox peaks that shift toward higher voltages with an increase in peak intensity. To investigate the charge storage mechanism for NH_4_^+^ ions, CV curves at different scan rates (5–50 mV s^−1^) are further analyzed. The total charge storage mechanism involves a capacitive (Faradaic) and diffusion‐controlled (non‐Faradaic) intercalation process. The relation between peak current versus scan rate is

(1) $$i=a\vartheta^{b}$$

(2) $$\log\left(i\right)=\log\left(a\right)+b\log\left(\vartheta \right)$$

where, the values of *a* is a coefficient and *b* determines whether the electrochemical reaction is controlled by diffu*s*ion (*b* = 0.5) or capaci*t*ive (*b* = 1) processes (Figure S5, Supporting Information). Figure 5b shows log–log plot between the peak current and scan rate. Using Dunn's equation *(i*_p_ *= K*_1_ϑ^0.5^ + K_2_ϑ), the contributions of the capacitive and the diffusive charge storage are quantified. The results show that charge storage is primarily driven by diffusive processes, accounting for ≈70%, with the remaining ≈30% controlled by capacitive processes, suggesting that the diffusive process dominates the electrochemical reaction and charge storage as illustrated in Figure 5c. The schematic diagram of the crystal structure during NH_4_^+^‐ion charging and discharging states is shown in Figure 5d,e, respectively. The presence of vacancies and filling of vacancies with NH_4_^+^ ions at the interstitial sites during the charging and discharging states can be visualized in the compound, where two active redox centers (Fe and Mn) govern the electrochemical performance of the compound. A schematic diagram of the aqueous NH_4_^+^‐ion battery based on KMnFeHCF as cathode and graphite as anode material is presented in Figure 5f. The CV curves recorded at different scan rates in 0.1 m (NH_4_)_2_SO_4_ electrolyte, along with the analysis of diffusive and capacitive contributions of KFeHCF compound are presented in the Figure S6 (Supporting Information).

**Figure 6:**
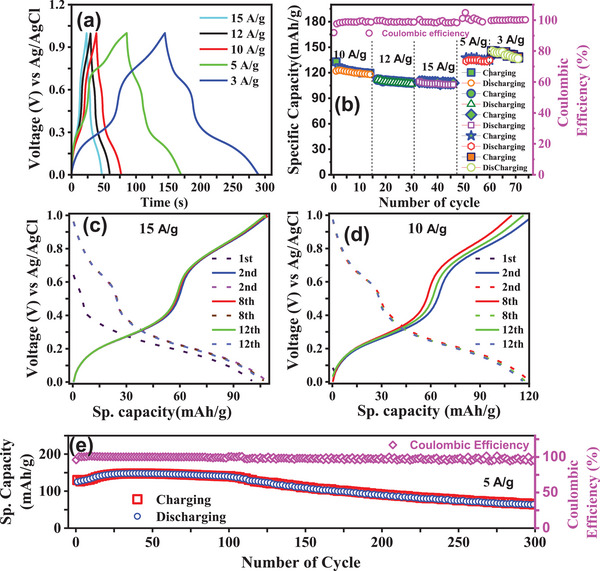
Electrochemical performance of KMnFeHCF in (NH_4_)_2_SO_4_ electrolyte. a) Voltage versus time profiles recorded at various current densities using GCD measurements. b) Specific capacity as a function of charge–discharge cycles at different current densities. Voltage versus specific capacity curves for multiple charge–discharge cycles at current densities of c) 15 A g^−1^, and d) 10 A g^−1^. e) Long‐term cycling stability and Coulombic efficiency at a current density of 5 A g^−1^.

**Figure** **6**a shows GCD measurement (voltage vs time response) at different current densities, indicating faster charging–discharging rates with increasing current densities. Specific capacity is calculated from these curves, and the relationship between specific capacity and charge densities for different number of charging–discharging cycles are shown in Figure 6b. The maximum specific capacity is found to be ≈145 mAh g^−1^ at 3 A g^−1^ current density. The consistency of specific capacity across cycles at each current density confirms the stable electrochemical performance of KMnFeHCF compound. Further confirmation of cyclic stability is provided by the voltage versus specific capacity data at 15 and 10 A g^−1^, as shown in Figure 6c,d. Discharge specific capacities of 105 mAh g^−1^ at 15 A g^−1^ and 120 mAh g^−1^ at 10 A g^−1^ are observed. The compound demonstrates exceptional electrochemical performance even at a high current density of 15 A g^−1^. Although, cathode material typically exhibits degradation upon GCD cycling at higher current densities, however, KMnFeHCF cathode material does not show any significant degradation, confirming structural stability of the compound. The charging and discharging specific capacity of KMnFeHCF as a function of number of cycles at 5 A g^−1^ shows 97% Coulombic efficiency, as shown in Figure 6e. For comparison, electrochemical performance in terms of voltage versus time, and voltage versus capacity of KFeHCF compound is shown in the Figure S7 (Supporting Information). The electrochemical results demonstrate that the specific capacity of KMnFeHCF is dominated by the redox‐active Mn along with larger channel size of PBAs. In KFeHCF, the channel size is ≈3.2 × 3.2 Å, which is larger than the ionic radius of NH_4_^+^ ion (1.48 Å). However, in the case of KMnFeHCF, the channel size for NH_4_^+^‐ion insertion expands due to smaller ionic radius of Mn, resulting in a higher storage capacity. However, excessive Mn doping could reduce the contribution of Fe to redox activity; therefore, an optimal balance of Mn and Fe concentrations is necessary to achieve maximum specific capacity. The comparison of the specific capacities of KMnFeHCF with reported PBAs in three‐electrode (half‐cell) configurations for AAIBs is presented in the Table S2 (Supporting Information). It shows that the KMnFeHCF's performance is highly competitive with materials like N‐HEPBA (a high‐entropy PBA),^[^ ^52^ ^]^ FeMnHCF,^[^ ^35^ ^]^ and MnPBA‐TA.^[^ ^53^ ^]^ The compound N‐HEPBA, operating in a 1 m (NH_4_)_2_SO_4_ + 5 mm CuSO_4_ electrolyte over a potential range of 0–1.2 V, delivers a specific capacity of 129 mAh g^−1^, however at a lower current density of 0.1 A g^−1^.^[^ ^52^ ^]^ While N‐HEPBA exhibits exceptional long‐term stability, maintaining ≈100% capacity retention after 1000 cycles, however, this work (on KMnFeHCF compound) achieves a higher capacity at a much higher current rate (145 mAh g^−1^ at 3 A g^−1^).

### 3.4 Study of KMnFeHCF Electrode for Aqueous AAIBs

**Figure 7:**
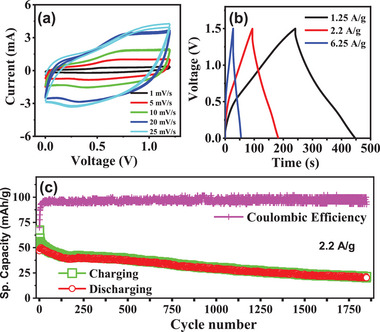
a) CV curves of the asymmetric two‐electrode aqueous cell at different scan rates. b) GCD curves at different current densities with a fixed voltage of 1.5 V. c) Specific capacity as a function of cycles of KMnFeHCF cathode.

A cathode using KMnFeHCF material is prepared and then investigated its electrochemical performances for AAIBs. The electrochemical properties of bare carbon cloth are also investigated to rule out its capacitive contribution (Figure S8, Supporting Information). **Figure** **7**a presents the CV curves (in two electrode setup) of the cathode electrode material recorded at various scan rates up to 1.2 V. Two broad redox peaks centered around 0.2 and 0.8 V are observed, corresponding to the Fe and Mn redox processes, respectively. While the overall shape of the CV curves remains consistent across scan rates, the peak currents increase with scan rate, indicating good rate capability. The CV curve at a fixed scan rate of 20 mV s^−1^ for various potential windows (Figure S9, Supporting Information), highlighting the optimal electrochemical stability of the electrode. Remarkably, the material sustains a wide potential window of up to 1.8 V without signs of electrolyte breakdown or material degradation. Figure 7b shows the GCD profiles at various current densities up to 1.5 V. The aqueous device delivers a specific capacity of 71 mAh g^−1^ at 1.25 A g^−1^ and 51 mAh g^−1^ at 2.2 A g^−1^ (Figure 7c). Impressively, the specific capacity remains high, retaining 50% of specific capacity after 1850 cycles, confirming its excellent rate performance and stability. It is to be noted that in the first few cycles, the capacity decreases rapidly due to the replacement of existing K⁺ ions, (located at interstitial sites) with NH_4_⁺ ions from the electrolyte. This occurs because the smaller K⁺ ions possess higher mobility compared to the larger, bulkier NH_4_⁺ ions. The energy dispersive X‐ray fluorescence data confirm that after 10 cycles, the interstitial K⁺ ions are completely replaced by NH_4_⁺ (Figure S10, Supporting Information). And after that, the electrochemical process proceeds solely through reversible NH_4_⁺ insertion/extraction, with K⁺ fully removed from the crystal structure.

**Figure 8:**
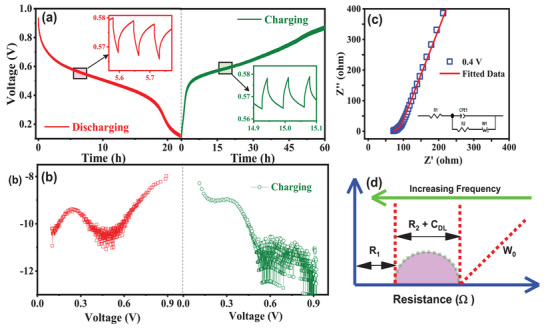
a) GITT curves showing the voltage versus time response for the KMnFeHCF electrode during discharging and charging cycles within a voltage range of 0.01–0.95 V. The inset highlights a magnified section of the GITT curve for both the discharging and charging processes. b) The diffusion coefficient of NH_4_⁺ ions during the discharging and charging cycles. c) EIS data presented as a Nyquist plot, recorded at 0.4 V for the NH_4_⁺‐ion aqueous battery electrode after GITT measurement. The inset shows the corresponding equivalent Randle's circuit. d) Schematic representation illustrating the various resistive elements and parameters involved in the Randle's circuit.

In addition, GITT measurement is also performed to explore the solid diffusion kinetics of NH_4_^+^ in KMnFeHCF cathode. **Figure** **8**a shows the results of the GITT applied to an aqueous NH_4_⁺‐ion battery, revealing the diffusion kinetics of NH_4_⁺ ions in a KMnFeHCF cathode. In this method, a constant current pulse of 13 mA g^−1^ is applied for 60 s to charge the cathode. This is followed by a 180 s rest period, allowing the system to stabilize and reach equilibrium at the open‐circuit potential. This cycle of current pulse followed by relaxation is repeated systematically over a voltage window of 0.01–0.95 V during both charging and discharging processes. The diffusion coefficient of NH_4_⁺ ions (*D*_NH4_^+^) is then derived using Fick's second law of diffusion, expressed as

(3) $$D=\frac{4}{\pi \tau }\times {\left(\frac{m_{B}V_{m}}{M_{B}S}\right)}^{2}\times {\left(\frac{\Delta E_{s}}{\Delta E_{t}}\right)}^{2}Valid\;for\;\tau <<\frac{L^{2}}{D_{NH4^{+}}}$$

where, *t* is the duration of the current pulse, *m*_B_ is the mass of the active cathode material, *V*_m_ is the molar volume, *M*_B_ is the molar mass, *S* is the geometric surface area (≈1 cm^2^), ∆*E*_s_ is the voltage change during the relaxation period, and ∆*E*_t_ is the voltage change during the current pulse. Figure 8b shows NH_4_^+^‐ion diffusion coefficient during discharging and charging cycles. The KMnFeHCF cathode has small values for both ∆*E*_t_ and ∆*E*_s_, suggesting a relatively low diffusion coefficient for NH_4_⁺ ions. At the beginning of the discharge process, *D*_NH4⁺_ of the KMnFeHCF is 7.98 × 10^−8^ cm^2^ s^−1^ (at 0.9 V), which decreases rapidly to a minimum value of 10.01 × 10^−11^ cm^2^ s^−1^ (at 0.47 V) at the plateau region. Afterward, the *D*_NH4⁺_ again increases to 9.38 × 10^−9^ cm^2^ s^−1^ (at 0.24 V) showing a notable V‐shaped profile.^[^ ^54^, ^55^ ^]^ For the charging cycle, the maximum value of *D*_NH4⁺_ is 8.28 × 10^−8^ cm^2^ s^−1^ (at 0.2 V), and a minimum value of 10.6× 10^−10^ cm^2^ s^−1^ (at 0.5 V) is observed at the plateau region. Afterward, diffusion coefficient decreases gradually with increasing potential, and remains nearly constant from 0.7 to 0.9 V. The electrochemical impedance spectroscopy (EIS) technique is employed to study the reaction kinetics of the KMnFeHCF cathode. EIS measurements for the KMnFeHCF cathode in a NH_4_⁺‐ion aqueous battery is presented in Figure 8c. The associated equivalent Randle's circuit (inset of Figure 8c) and a schematic depiction of internal resistance elements is shown in the Figure 8d. The KMnFeHCF electrode exhibits a low charge transfer resistance (*R*_2_) of around ≈3Ω, reflecting fast NH_4_⁺‐ion transport kinetics. The EIS data of KFeHCF and KMnFeHCF films using three electrode setup are presented in the Figures S11 and S12 (Supporting Information), respectively.

To gain insights into the NH_4_⁺‐ion storage mechanism and the corresponding structural evolution of the cathode material, comprehensive analysis using ex situ XRD, XPS, and Mössbauer spectroscopy techniques are performed at different states of charge and discharge cycle. The XPS study reveals changes in the chemical states of Fe and Mn atoms during NH_4_⁺‐ion insertion and extraction. Whereas, Mössbauer spectroscopy study provides information on HS/LS‐Fe^+2^/Fe^+3^ ions and their respective ratio during NH_4_⁺‐ion insertion and extraction. In addition, variation within the local environment surrounding the Fe atoms throughout charge and discharge cycle is also revealed.

**Figure 9:**
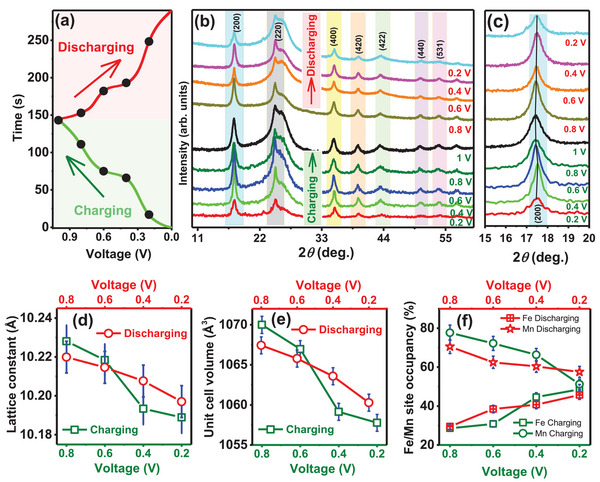
Investigation of the NH_4_^+^‐ion storage mechanism. a) Charge/discharge profile of the cathode electrode material at a current density of 3 A g^−1^ over a voltage window of 0–1 V. b) Ex situ XRD patterns recorded at various voltages (indicated by black dots in panel (a)) during the charge/discharge cycle. c) Enlarged view of the (200) Bragg peak in the XRD patterns at different charge/discharge voltages. Variation of d) lattice constant, e) unit cell volume, and f) Fe/Mn site occupancies during charging–discharging cycle.

The XRD measurements are recorded at different voltages marked on the charge/discharge curve, recorded at a current density of 3 A g^−1^, shown in **Figure** **9**a. The XRD patterns, shown in Figure 9b reveal distinct diffraction Bragg peaks located at 17.4°, 24.8°, and 35.2°, corresponding to the (200), (220), and (400) planes, respectively of a fcc crystal structure. These diffraction peaks remain clearly visible and unchanged throughout the charge/discharge cycle, indicating that the cathode material retains its cubic framework structure and demonstrates excellent structural stability during NH_4_⁺‐ion insertion and extraction. An enlarged view of the most intense (200) Bragg peak is shown in the Figure 9c, showing that the peak position shifts to lower 2*θ* during the charging process and returns to its original position during discharging. This behavior signifies lattice expansion during NH_4_⁺‐ion insertion and lattice contraction during NH_4_⁺‐ion extraction. In addition, the Rietveld refinement of room temperature powder XRD pattern of KMnFeHCF electrode at various states of charging and discharging is presented in Figure S13 (Supporting Information). The lattice constant, unit cell volume, and Fe/Mn site occupancy at charging/discharging voltages are extracted from the Rietveld refined XRD data and are presented in Figure 9d–f, respectively. A small increase in lattice constant (≈0.4%) (Figure 9d) and unit cell volume (≈1.3%) (Figure 9e) is observed during the charging process, which reverses upon discharging, confirming a robust and stable framework crystal structure for NH_4_⁺‐ion insertion and extraction. Interestingly, Fe site occupancy decreases, while Mn site occupancy increases during the charging and discharging cycle (Figure 9e), suggesting the active involvement of Mn in the charge storage process, which contributes to the enhanced specific capacity of the compound.

**Figure 10:**
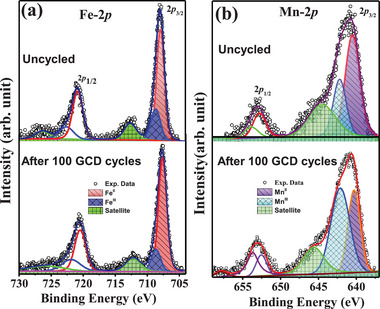
a) Fe‐2p and b) Mn‐2p core level XPS spectra of KMnFeHCF electrode for uncycled and after 100 GCD cycles.

**Figure** **10**a shows the Fe‐2p core‐level spectra for the uncycled and cycled (100 GCD) KMnFeHCF electrode. Two main peaks, separated by 13 eV are observed, originating from spin–orbit coupling and corresponding to the 2p_3/2_ and 2p_1/2_ transitions, along with noticeable satellite features. The 2p_3/2_ region is deconvoluted into two peaks at 708 and 709.5 eV, confirming the presence of Fe⁺^2^ and Fe⁺^3^ oxidation states. For the pure metallic Fe, the core level peak is found to be at 707.0 eV which is shifted to higher binding energy side after coordinating with ions of different electronegativity.^[^ ^56^ ^]^ For K_4_[Fe(CN)_6_] compound, the binding energy position of Fe^+2^ lies in between 707.4 and 708.6 eV, whereas Fe^+3^ in K_3_[Fe(CN)_6_] is found to be around 709.5–710 eV.^[^ ^57^ ^]^ The ratio of the Fe⁺^2^ and Fe⁺^3^ ions does not change much in case of uncycled and cycled (100 GCD) KMnFeHCF cathode. The fitted core‐level spectra of Mn‐2p for the KMnFeHCF electrode, both uncycled and after 100 GCD cycles, are shown in Figure 10b. Two main peaks are observed at 653 and 641.0 eV, corresponding to Mn‐2p_3/2_ and 2p_1/2_ transitions, respectively, with a spin–orbit splitting of 12 eV. The asymmetry in the Mn‐2p peaks indicates the presence of mixed‐valence manganese. The Mn 2p_3/2_ peak is deconvoluted into two peaks located at 640.5 and 642.1 eV. Specifically, the peak at 640.5 eV is attributed to Mn^+2^, while the peak at 642.1 eV corresponds to Mn^+3^.^[^ ^58^ ^]^ The amount of Mn^+2^ ions is found to be slightly higher than that of Mn^+3^ ions in uncycled KMnFeHCF cathode, whereas after 100 GCD cycle, the amount of Mn^+3^ ions becomes more than that of Mn^+2^ ions. XPS results corroborate the XRD observation that Mn is actively involved in charge storage process, thus significantly enhancing the specific capacity of the compound. The peak at 644.6 eV is designated as the satellite peak.^[^ ^59^ ^]^ C‐1s spectra (Figure S14, Supporting Information) is deconvoluted for three peaks positioned at 284.8, 285.8, and 287.9 eV. The peak at 284.8 eV is originated from adventitious carbon and usually taken as charge reference. The medium intensity peak at 285.8 eV has been assigned to sp^2^ carbon–nitrogen bonding. The broad peak observed at 287.9 eV is ascribed to the π → π* transition in the cyanide ligand.^[^ ^60^ ^]^ The N‐1s peak is sensitive for small changes that occur when the C≡N ligand is coordinated to any metal ion to form the complex. For example, N‐1s peak appears at 398 eV in KCN, however, shows a negative shift of binding energy −0.7 and −0.5 eV for K_4_[Fe(CN)_6_]·3H_2_O and K_3_[Fe(CN)_6_], respectively. The N‐1s peak (Figure S14, Supporting Information) is fitted with three deconvoluted peaks at 397.5, 399.1, and 402.5 eV.^[^ ^61^ ^]^ The peak at 397.5 eV is assigned to nitrogen of C≡N ligand.^[^ ^62^ ^]^ Whereas, the peak at 399.1 eV for Fe^+3^─C≡N and 402 eV for oxidized N and 402.5 eV can be attributed to charge transfer process, and positive charged N in the compound.^[^ ^63^, ^64^, ^65^ ^]^ It is reported that the peak at 399.1 eV can be attributed to C═N─C bond, whereas, the peak at 402.5 eV may be assigned to N─O bond.^[^ ^66^ ^]^ Moreover, the peak at 402.5 eV may also indicate the presence of hydrogen‐bonded nitrogen also.^[^ ^63^, ^67^ ^]^ The O‐1s spectrum (Figure S14, Supporting Information) exhibited a broad peak cantered at 531.9 eV, which can be attributed to ─OH from the crystal water.^[^ ^68^ ^]^ The O‐1s peak is deconvoluted into three peaks at 531, 532.4, and 533.5 eV assigned to the oxygen of OH group, H_2_O, and C═O group.^[^ ^69^, ^70^ ^]^ The XPS results are consistent with the Mössbauer results which also confirm the presence of Fe in LS‐Fe^+2^ and HS‐Fe^+3^ oxidation state.

**Figure 11:**
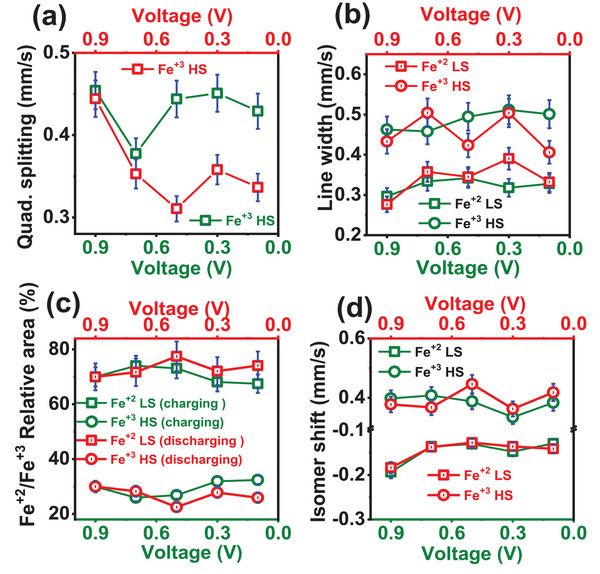
Variation of hyperfine parameters at different states of charging and discharging: a) quadrupole splitting, b) line width, c) ratio of Fe⁺^2^‐LS to Fe⁺^3^‐HS, and d) isomer shift. The electrode is charged from 0.1 to 1.0 V and then discharged back to 0.1 V. The line width, quadrupole splitting, and isomer shift value for 0.9 V charged electrode is shown for singlet and doublet A.

Ex situ Mössbauer spectroscopy (Figure S15, Supporting Information) is carried out at different stages of charging and discharging of electrode to study the variation in a relative % of Fe^+2^‐LS and Fe^+3^‐HS ions and their quadrupole splitting which is associated to the distortion present in the compound. The spectra are fitted with singlet and a doublet. The values of fitted parameters are given in the Table S1 (Supporting Information). The quadrupole splitting value of the Fe⁺^3^ HS doublet increases during charging, indicating enhanced local distortion associated with NH_4_⁺‐ion insertion up to 0.5 V. However, once the NH_4_⁺ ions are fully inserted into the structure, the distortion appears to stabilize, showing reduced variation, as observed in **Figure** **11**a. By contrast, during the extraction (discharging) process, the quadrupole splitting decreases, suggesting a minimum distortion as NH_4_⁺ ions leave the structure. Notably, the distortion reaches a maximum around 0.3 V during discharging, which likely corresponds to the point at which NH_4_⁺ ions begin to vacate the structure. These observations suggest that 0.7 V during charging and 0.3 V during discharging represent the critical voltages where NH_4_⁺ ions are fully inserted into or completely extracted from the 8c crystallographic sites, respectively, indicating full occupation and vacancy transitions. As shown in Figure 11b, the line widths of both Fe⁺^2^ LS and Fe⁺^3^ HS components decrease during the charging process. However, during discharging, the line width of the Fe⁺^3^ HS state remains nearly constant, whereas the line width of the Fe⁺^2^ LS state increases, indicating a possible change in the local environment or electronic configuration associated with the NH_4_⁺‐ion extraction. The relative percentage of Fe⁺^2^ LS ions increases, while that of Fe⁺^3^ HS ions decreases during charging up to 0.7 V, indicating a reduction of Fe⁺^3^ HS to Fe⁺^2^ LS as NH_4_⁺ ions are inserted. At 0.9 V, the Mössbauer spectrum is best fitted with one singlet and three doublets, suggesting changes in the local environments of both Fe⁺^2^ and Fe⁺^3^ HS ions, possibly due to structural distortion or coordination variation. The variation in the relative proportions of Fe⁺^2^ and Fe⁺^3^ ions during charging and discharging is shown in Figure 11c. Additionally, the isomer shift (Figure 11d) of the Fe⁺^3^ ion increases with charging, implying an elongation of the Fe⁺^3^─C bond length, consistent with lattice expansion during NH_4_⁺ insertion.

**Figure 12:**
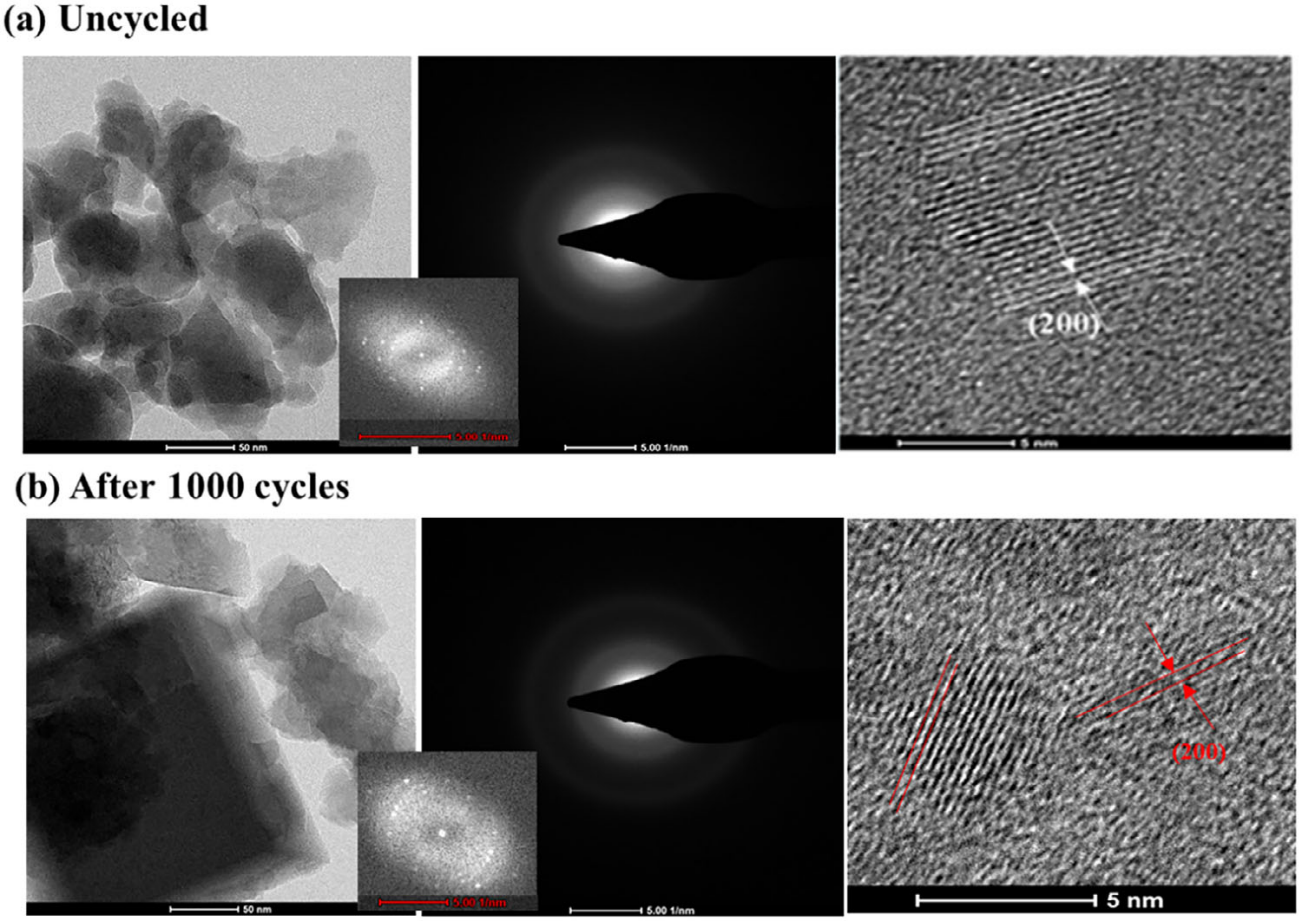
HRTEM images of KMnFeHCF material in the a) uncycled state and b) after 100 GCD cycles. The insets show the fast Fourier transform (FFT) patterns of the selected area electron diffraction (SAED).

The microstructure of the electrode material is also investigated for uncycled and cycled cathode materials using HRTEM. The **Figure** **12**a shows HRTEM image of the uncycled compound revealing uniformly distributed fine particles with high degree of crystallinity as evidenced by the presence of distinct lattice fringes. A clearly defined (200) plane with an interplanar spacing of ≈4.9 Å further confirms the structural integrity and crystalline nature of the material prior to cycling. After 1000 charge–discharge cycles, the HRTEM image in Figure 12b shows that the microstructural remains stable. The continued presence of clear lattice fringes suggests that the open framework structure retain its stability even after extended GCD cycling. The observed lattice planes exhibit a *d*‐spacing of ≈4.7 Å, confirming the preservation of crystallinity postcycling.

**Figure 13:**
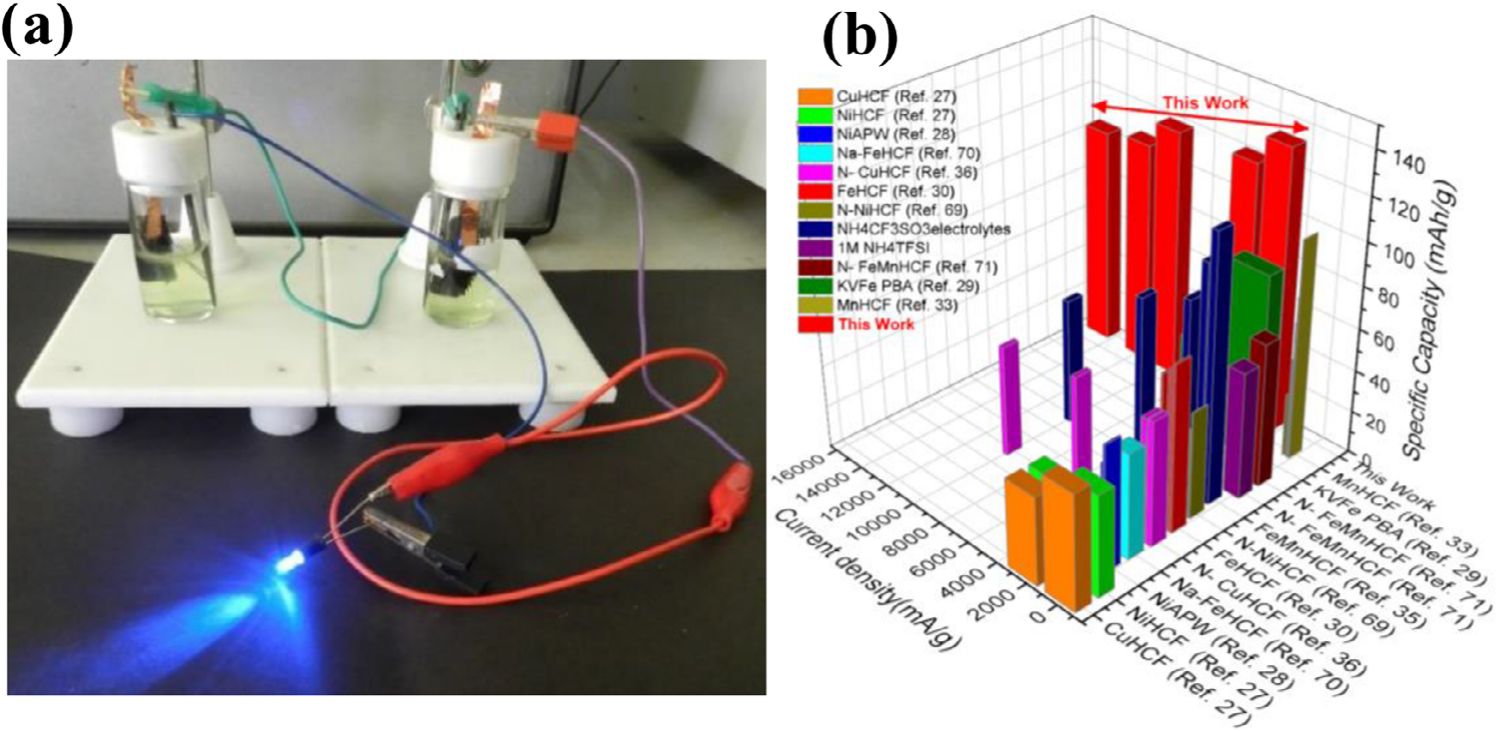
a) Demonstration of a LED powered by an assembly of two aqueous NH_4_^+^‐ion asymmetric full cells using the KMnFeHCF cathode. b) Comparison of the specific capacity of the KMnFeHCF compound with those of other PBAs reported in the literature.

To assess the practical applicability of KMnFeHCF cathode, an asymmetric aqueous full cell is assembled and tested using 1 m (NH_4_)_2_SO_4_ as the electrolyte. **Figure** **13**a shows a photograph of the assembled device successfully powering a LED light, confirming the material's feasibility for real‐world applications. The device achieved a maximum operating voltage of ≈2.5 V, with a specific capacity of 71 mAh g^−1^. The video demonstrating the LED powered by an assembly of two aqueous NH_4_^+^‐ion asymmetric full cells using the KMnFeHCF cathode is presented as Movie M1 (Supporting Information). The specific capacity of the KMnFeHCF cathode is compared with those of other PBAs reported in the literature for AIBs, as shown in Figure 13b. The KMnFeHCF material demonstrates superior specific capacity, indicating its excellent electrochemical performance (Table S3, Supporting Information) relative to previously reported PBA‐based cathodes for aqueous AAIBs.^[^ ^71^, ^72^, ^73^ ^]^

The KMnFeHCF//graphite full cell demonstrates strong competitiveness among AAIBs, particularly in terms of power density and cycling stability. Although its energy density is lower than the systems like PTPD//CuFe‐PBA (206.6 mAh g^−1^)^[^ ^74^ ^]^ and FeMnHCF//PTCDI (123.8 mAh g^−1^)^[^ ^35^ ^]^ both measured at low current density of 0.5 A g^−1^ (Table S3, Supporting Information). However, KFeMnHCF delivers a specific capacity of ≈71 mAh g^−1^ even at a higher current density of 1.25 A g^−1^. Moreover, KMnFeHCF achieves a significantly higher power density of 1103 W kg^−1^ (vs 502.38 W kg^−1^ for the PTPD//CuFe‐PBA). The superior power output, combined with high‐rate capacity highlights remarkable promise as a robust cathode for AAIBs.

### 3.5 DFT Analysis of NH_4_^+^‐Ion Storage Mechanism in KMnFeHCF

**Figure 14:**
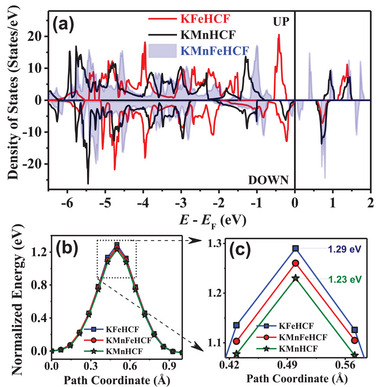
a) Comparison of the DOS of KFeHCF, KMnFeHCF, and KMnHCF compounds. b) Calculated energy barriers for NH_4_⁺‐ion migration between two 8c crystallographic sites via the 24d site in all three compounds. c) Magnified view of the marked region of (b) is shown.

**Figure** **14**a shows the electronic density of states (DOS) of KFeHCF, KMnFeHCF, and KMnHCF compounds. The DOS of KMnFeHCF is different from its single‐metal counterparts (FeHCF, MnHCF), underscoring the importance of Mn incorporation. KFeHCF shows a bandgap of 1.3 (majority spins) and 0.6 (minority spins) eV, dominated by localized Fe 3d states hybridized with cyanide ligands. Simillarly, for Mn⁺^2^ (HS d⁵) in KMnHCF a bandgap of ≈0.6 eV, for spin‐down states, found dominating near the Fermi level. KMnFeHCF, however, presents a hybrid electronic structure rather than a simple average, where Mn spin‐down states dominate and generate new hybridized levels near the Fermi energy. This enhances electron delocalization, improves bulk conductivity, and enables efficient charge compensation during NH_4_⁺ intercalation/deintercalation. PDOS analysis (Figure S16, Supporting Information) further confirms that the Mn⁺^2^/⁺^3^ redox couple operates at a higher potential than Fe⁺^3^/⁺^2^, explaining the separated redox peaks and stable voltage plateaus. Importantly, rational tuning of Fe/Mn ratios mitigates the Jahn–Teller distortions observed in MnFeHCF, ensuring structural stability and long‐term cyclability. Mn incorporation also critically influences ion transport. KFeHCF shows a high NH_4_⁺ migration barrier of 1.29 eV, while KMnFeHCF exhibits a reduced barrier of 1.26 eV with a flatter diffusion pathway (Figure 14b,c). This improvement arises from i) lattice expansion due to the larger Mn⁺^2^ ionic radius (≈0.83 Å vs Fe⁺^2^ ≈0.78 Å), which increases the unit cell from ≈10.10 to 10.19 Å and widens NH_4_⁺ (≈1.43 Å) diffusion channels, and ii) enhanced local flexibility, as Mn─N bonds elongate (1.918 → 1.925 Å) upon NH_4_⁺ insertion. These structural adaptations reduce the energetic cost of ion hopping, enabling faster diffusion, higher rate capability, and robust electrochemical performance.

A deeper mechanistic insight of NH_4_^+^‐ion storage mechanism is systematically investigated by DFT calculations. The NH_4_^+^‐ion storage in the PB framework material is analyzed theoretically by rotating the NH_4_^+^ around N atom in all possible ways. Beside these rotations, the study was also carried out by considering the translation motion of NH_4_^+^ from one 8c crystallographic site to another 8c site though 24d site. The most stable configuration is obtained when NH_4_^+^ ion is at 8c site, as this site provides the largest free space in comparison with other sites with minimum structural deformation.

**Figure 15:**
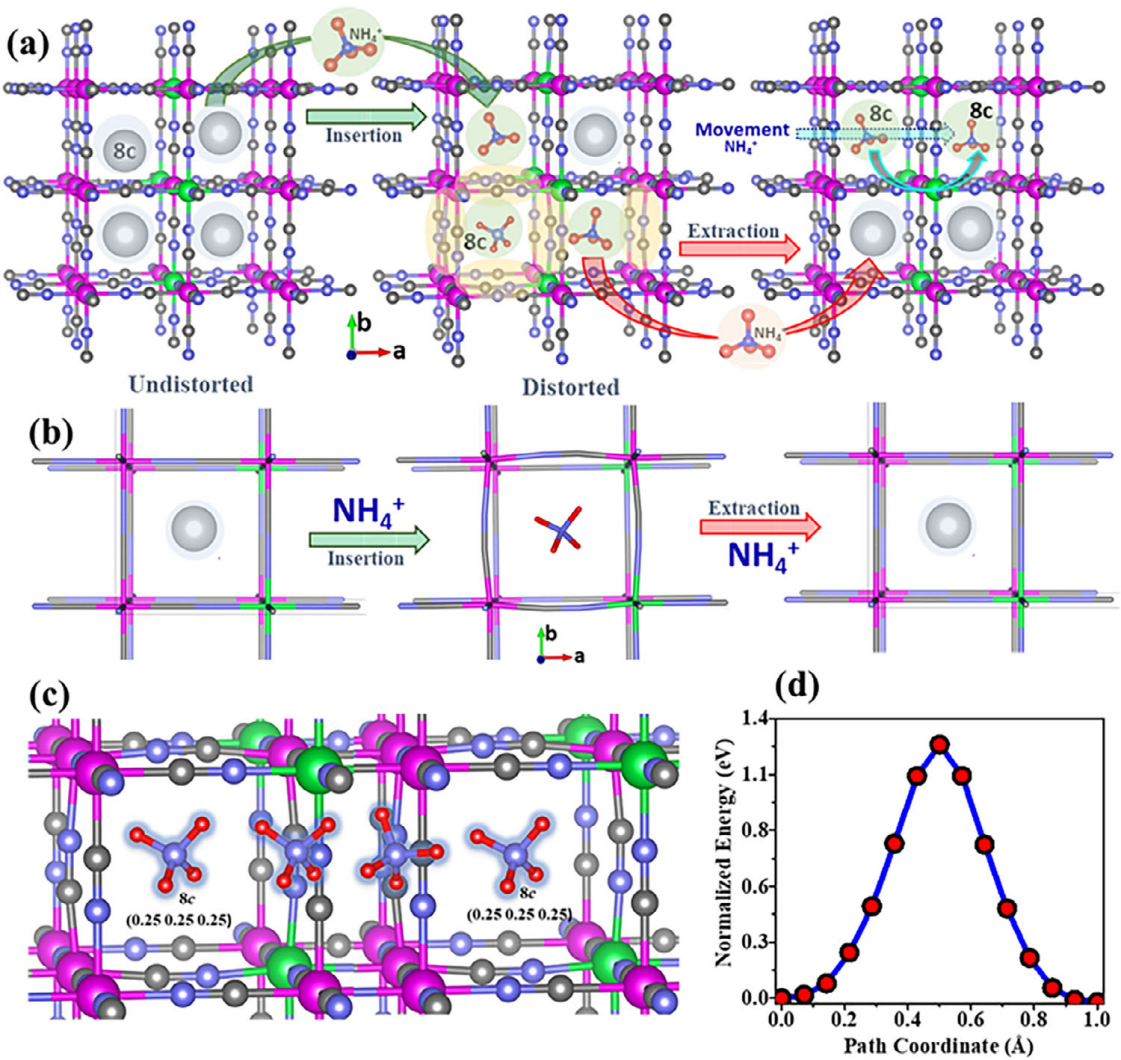
a) Schematic representation of NH_4_⁺‐ion insertion and extraction in the cubic open‐framework crystal structure of the KMnFeHCF compound. b) DFT calculations showing the presence and absence of NH_4_⁺ ions during insertion and extraction result in distorted and undistorted crystal structures, respectively. c) Translation motion of NH_4_⁺ ions and d) calculated energy barrier for migration of the NH_4_⁺ ion between two 8c crystallographic sites via the 24d site in the KMnFeHCF open framework.

**Figure** **15**a shows a schematic diagram of NH_4_⁺‐ion insertion and extraction within the crystal structure of the KMnFeHCF, derived from DFT calculations. The structure features an open tunnel framework with vacant interstitial 8c crystallographic sites, which serve as favorable hosts for NH_4_⁺ ions. In the absence of NH_4_⁺‐ion insertion, the unit cell remains undistorted, as depicted in Figure 15b. In this uncycled state, the lattice constant is found to be 10.16 Å, and the Fe─C/N and Mn─C/N bond lengths are ≈2.46 and 2.21 Å, respectively. However, upon NH_4_⁺‐ion insertion at the 8c sites, structural distortion is observed. The Fe─C/N and Mn─C/N bond lengths slightly increase to 2.48 and 2.25 Å, respectively. Despite this distortion, the compound maintains structural integrity, demonstrating sufficient flexibility to accommodate the strain without forming to a secondary phase or experiencing structural collapse. Upon NH_4_⁺‐ion extraction, the structure returns to its original undistorted cubic structure. This behavior is further supported by ex situ XRD studies, which reveal a reversible increase in unit cell volume during the insertion and extraction of NH_4_⁺ ions throughout the charge–discharge process. A movie based on DFT calculations, demonstrating how strain leads to bond length distortion in the crystal structure's unit cell during NH_4_⁺‐ion insertion, is provided in Movie M2 (Supporting Information). Figure 15c illustrates the translational motion of the NH_4_⁺ ion from one 8c crystallographic site to another via the 24d site, along with the associated energy barrier shown in Figure 15d. The symmetric energy profile exhibits a maximum barrier of ≈1.26 eV, representing the activation energy for ion diffusion. Although slightly higher than for smaller ions like Li⁺ or Na⁺, however, this barrier energy is still within a feasible range for effective operation under standard battery conditions. These calculations confirm the structural compatibility and favorable electrochemical performance of KMnFeHCF as a cathode material for AAIBs.

In summary, the structural, magnetic, and electrochemical properties of KMnFeHCF have been comprehensively investigated and demonstrated its exceptional potential as an ultrafast, high‐performance advanced cathode material for AAIBs. The experimental findings are further supported with theoretical models using DFT, validate the material's versatile functionality. The incorporation of manganese provides additional redox‐active sites, which markedly enhance charge storage capacity and cycling stability. Importantly, KMnFeHCF exhibits high specific capacity, ultrafast charging, and outstanding long‐term stability, even at elevated current densities. These results highlight KMnFeHCF as a cost‐effective, multifunctional cathode candidate, paving the way for the development of advanced, metal‐free AAIB technologies.

## 4 Conclusion

This study presents KMnFeHCF, a PBA molecular magnet synthesized via the coprecipitation method, as a promising cathode material for metal‐free AAIB. KMnFeHCF was extensively characterized for its structural, magnetic, and electrochemical properties, and correlations among these were established. XRD confirms a fcc structure (*Fm*3*m*) with a lattice parameter of ≈10.19 Å. Fe─C (≈1.96 Å) and Fe─N (≈1.85 Å) bond lengths support the cyanide‐bridged framework's stability. Mössbauer and magnetization data confirm mixed Fe⁺^2^/Fe⁺^3^ valence and weak ferromagnetic behavior, while XPS reveals the coexistence of Mn⁺^2^/Mn⁺^3^ and Fe⁺^2^/Fe⁺^3^ redox states. HS Mn⁺^3^ ions induce Jahn–Teller distortions that impede electron transfer and slow redox kinetics, whereas LS Fe⁺^3^/Fe⁺^2^ states facilitate faster and more reversible charge transfer demonstrating how spin states govern the electrochemical behavior of KMnFeHCF. Electrochemical studies show a high specific capacity of ≈145 mAh g^−1^ at 3 A g^−1^ and ≈130 mAh g^−1^ at 5 A g^−1^, with 97% Coulombic efficiency and  retention of the same over 300 cycles. In full‐cell tests, specific capacities of 71 mAh g^−1^ at 1.25 A g^−1^ and 51 mAh g^−1^ at 2.2 A g^−1^ were achieved, retaining 50% of initial capacity after 1850 cycles. GITT profiles exhibit V‐shaped diffusion behavior, with diffusion coefficients up to 7.98 × 10^−8^ cm^2^ s^−1^ (discharge) and 8.28 × 10^−8^ cm^2^ s^−1^ (charge). Ex situ XRD, XPS, and Mössbauer reveal reversible lattice strain and Fe HS/LS changes during cycling, without structural collapse. DFT calculations support low NH_4_⁺ migration barriers (≈1.2 eV) and stable ion insertion. Even though the loading amount (≈1 mg cm^−^^2^) is relatively low, a full cell using KMnFeHCF/graphite confirms its practical viability. With high capacity, redox stability, and eco‐friendly composition, KMnFeHCF is a strong candidate for next‐generation AAIBs.

## Conflict of Interest

The authors declare no conflict of interest.

## Supporting information



Supporting Information

Supplemental Movie 1

Supplemental Movie 2

## Data Availability

The data that support the findings of this study are available from the corresponding author upon reasonable request.
