# Supplementary material for: An Advanced High‐Performance Ultrafast Ammonium‐Ion Aqueous Battery Based on Dual‐Metal Redox Open Framework Molecular Magnet
Source: Adv Sci (Weinh). 2026 Jan 15;13(12):e14287. doi: 10.1002/advs.202514287 (PMC12948221; doi:10.1002/advs.202514287)
Supplement: Supplementary file 1 — Supporting Information [file ADVS-13-e14287-s003.docx]

**An Advanced High-Performance Ultra-Fast Ammonium-Ion Aqueous Battery Based on Dual-Metal Redox Open Framework Molecular Magnet**

**Nilasha Maiti^1,2^, Pramod Bhatt^1,2, *^, M K Sharma^2,3^, M D Mukadam^1^, Sher Singh Meena^1^, Soumen Samanta^2,4^**

*^1^Solid State Physics Division, Bhabha Atomic Research Centre, Mumbai 400 085, India*

^2^*Homi Bhabha National Institute, Anushaktinagar, Mumbai 400 094, India*

*^3^Fuel Chemistry Division, Bhabha Atomic Research Centre, Mumbai 400 085, India*

*^4^Technical Physics Division, Bhabha Atomic Research Centre, Mumbai 400 085, India*

**Supporting Information**

**Figure S1**: (a) Electrochemical film deposition of KFeHCF and (b) KMnFeHCF compounds using cyclic voltammetry.

**
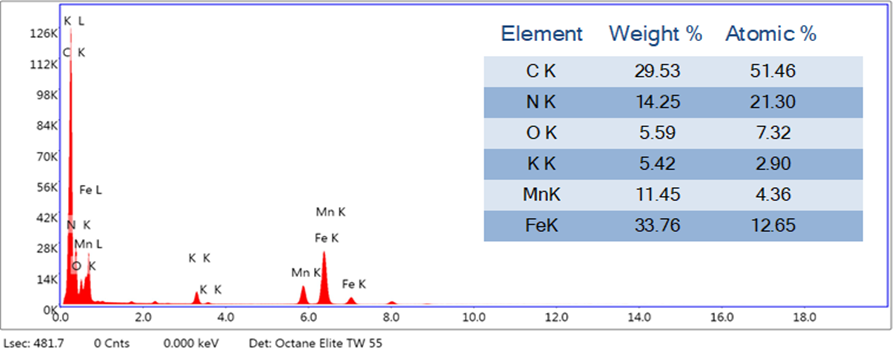
**

**
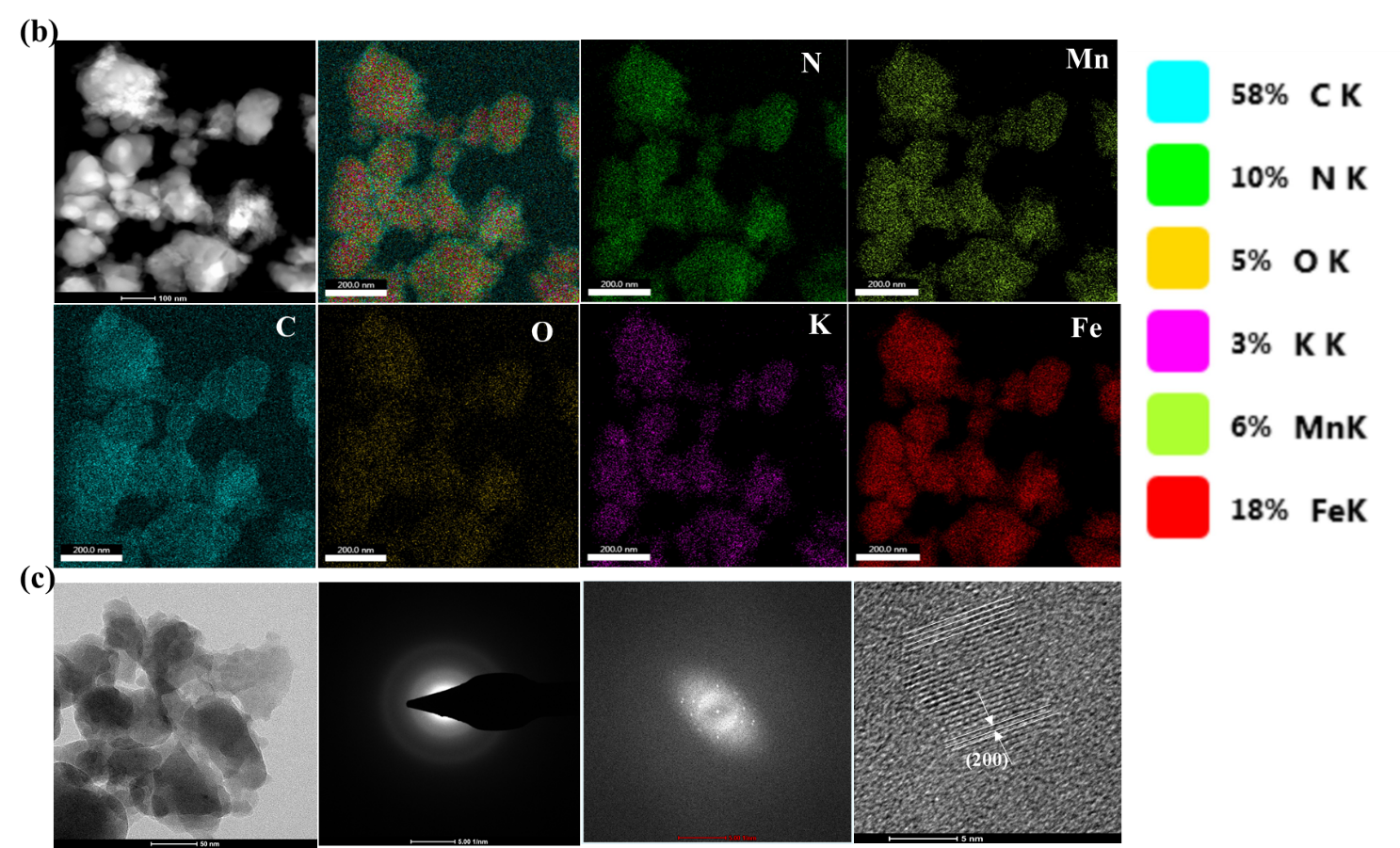
**

**Figure S2:** Energy Dispersive X-ray Spectroscopy (EDS) elemental mapping of the KMnFeHCF compound. The special distribution of the elemental mapping confirms the uniform distribution of the individual metals in the compound.

**Figure S3:** Fitted room temperature Mössbauer spectra of various compounds. The recorded experimental data (open circles) is fitted with singlet and doublet shown by the thick green and blue lines, respectively. Inset shows spectra up to velocity ±4 mm/s.

**Table S1:** Hyperfine parameters determined by fitting the experimental room-temperature Mössbauer spectra of the compounds.

| Samples | Fe-sites  and ionic states | Isomer Shift  (δ) (mm/s)  ± 0.008 | Quadrupole splitting  (ΔE_Q_) (mm/s)  ± 0.005 | Relative area (%) | Line width  (Γ) (mm/s)  ± 0.005 | Goodness of fit  (ꭓ^2^) |
| --- | --- | --- | --- | --- | --- | --- |
| Fe(SO_4_) | Doublet  (Fe^+2^ HS) | 1.26 | 3.236 | 100 | 0.314 | 1.07 |
| Fe_2_(SO_4_)_3_ | Doublet A  (Fe^+3^ HS) | 0.37724 | 0.367 | 65.5 | 0.354 | 1.06 |
|  | Doublet B  (Fe^+3^ HS) | 0.53736 | 0.353 | 34.5 | 0.287 |  |
| K_3_[FeCN_6_] | Doublet  (Fe^+3^-LS) | -0.146 | 0.253 | 80.4 | 0.253 | 0.901 |
|  |  | -0.094 | 0.293 | 19.6 | 0.230 |  |
| KFeHCF | Singlet  (Fe^+2^-LS) | -0.156 | - | 42.8 | 0.299 | 0.86 |
|  | Doublet  (Fe^+3^ HS) | 0.382 | 0.509 | 57.1 | 0.454 |  |
| KMnFeHCF | Singlet  (Fe^+2^-LS) | -0.160 | -- | 64.4 | 0.315 | 1.07 |
|  | Doublet  (Fe^+3^ HS) | 0.379 | 0.527 | 35.5 | 0.452 |  |
| Charge  0.1 V | Singlet  (Fe^+2^-LS) | -0.129 | -- | 67.5 | 0.328 | 0.90 |
|  | Doublet  (Fe^+3^ HS) | 0.383 | 0.429 | 32.4 | 0.501 |  |
| 0.3 V | Singlet  (Fe^+2^-LS) | -0.147 | -- | 68.1 | 0.318 | 1.04 |
|  | Doublet  (Fe^+3^ HS) | 0.335 | 0.451 | 31.9 | 0.512 |  |
| 0.5 V | Singlet  (Fe^+2^-LS) | -0.131 | -- | 73.1 | 0.342 | 0.93 |
|  | Doublet  (Fe^+3^ HS) | 0.388 | 0.444 | 26.9 | 0.495 |  |
| 0.7 V | Singlet  (Fe^+2^-LS) | -0.136 | -- | 74.0 | 0.334 | 0.93 |
|  | Doublet  (Fe^+3^ HS) | 0.408 | 0.377 | 25.9 | 0.458 |  |
| 0.9 V | Singlet  (Fe^+2^-LS) | -0.194 | -- | 53.8 | 0.297 | 0.92 |
|  | Doublet A  (Fe^+3^ HS) | 0.397 | 0.627 | 15.8 | 0.293 |  |
|  | Doublet B  (Fe^+2^-HS) | 0.215 | 0.587 | 16.2 | 0.250 |  |
|  | Doublet C  (Fe^+3^ LS) | 0.099 | 0.331 | 14.1 | 0.286 |  |
| Discharge  0.9 V | Singlet  (Fe^+2^-LS) | -0.184 | -- | 70 | 0.277 | 1.02 |
|  | Doublet  (Fe^+3^ HS) | 0.377 | 0.404 | 30 | 0.433 |  |
| 0.7 V | Singlet  (Fe^+2^-LS) | -0.137 | -- | 71.7 | 0.358 | 0.85 |
|  | Doublet  (Fe^+3^ HS) | 0.368 | 0.353 | 28.3 | 0.504 |  |
| 0.5 V | Singlet  (Fe^+2^-LS) | -0.127 | -- | 77.5 | 0.345 | 0.91 |
|  | Doublet  (Fe^+3^ HS) | 0.446 | 0.3105 | 22.5 | 0.424 |  |
| 0.3 V | Singlet  (Fe^+2^-LS) | -0.136 | -- | 72.1 | 0.390 | 0.95 |
|  | Doublet  (Fe^+3^ HS) | 0.362 | 0.358 | 27.8 | 0.504 |  |
| - 1. V | Singlet  (Fe^+2^-LS) | -0.141 | -- | 74.1 | 0.332 | 0.89 |
|  | Doublet  (Fe^+3^ HS) | 0.418 | 0.336 | 25.9 | 0.406 |  |

**Figure S4:** CV curves recorded for KMnFeHCF compound at a scan rate of 10 mV/s in 0.1 *M* (NH_4_)_2_SO_4_ electrolyte.

**
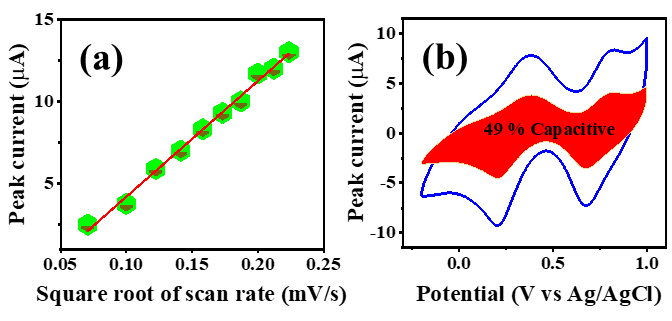
**

**Figure S5**: The peak current versus the square root of scan rate (a) and peak current versus voltage (b) KMnFeHCF compound at 50 mV/s.

# Figure S6: (a) CV curves of KFeHCF film in 0.1 *M* (NH_4_)_2_SO_4_ electrolyte at different scan rates. Inset of Fig. (a) shows log-log plot between the peak current and scan rates. (b) Diffusion (non-Faradic) and Faradic (capacitive) controlled capacities at different scan rates.

**Figure S7**: (a) voltage vs. time response of KFeHCF film at different current densities. (b) The specific capacity as function of cycles at different current densities. Various cycles of charging and discharging in term of voltage vs. specific capacity at 10 A/g (c), and 20 A/g (d) exhibit constant rate capacities.

**Figure S8:** Electrochemical characterization of bare carbon cloth and its comparison with KMnFeHCF deposited on carbon cloth (KMnFeHCF/carbon cloth)

**Figure S9**: (a) CV curves of the asymmetric two-electrode aqueous cell at a fixed scan rate of 20 mV/s for varying potential windows. GCD curves of the two-electrode cell at a fixed current

density of 2.2 A/g with varying voltage windows (b). Voltage vs specific capacity (c) and specific capacity as a function of current density (d) of KMnFeHCF cathode.

**

**Figure S10:** The Energy Dispersive X-ray Fluorescence (EDXRF) data of KMnFeHCF electrode before and after 10 charging-discharging cycles.

**Figure S11:** (a) Electrochemical impedance spectroscopy (EIS) data of KFeHCF film in ammoniated states. (b) The relationship between Z′ and ω^-1/2^ at low frequency of KFeHCF film.

**Figure S12:** (a) Electrochemical impedance spectroscopy (EIS) data of KMnFeHCF compound in ammoniated states. The inset of (a) shows equivalent Randle’s circuit. (b) The relationship between Z′ and ω^-1/2^ at low frequency of KMnFeHCF film.

**Figure S13**: Rietveld refinement of Room temperature powder XRD pattern of KMnFeHCF electrode at various voltages of charging and discharging. XRD pattern of carbon cloth is also shown.


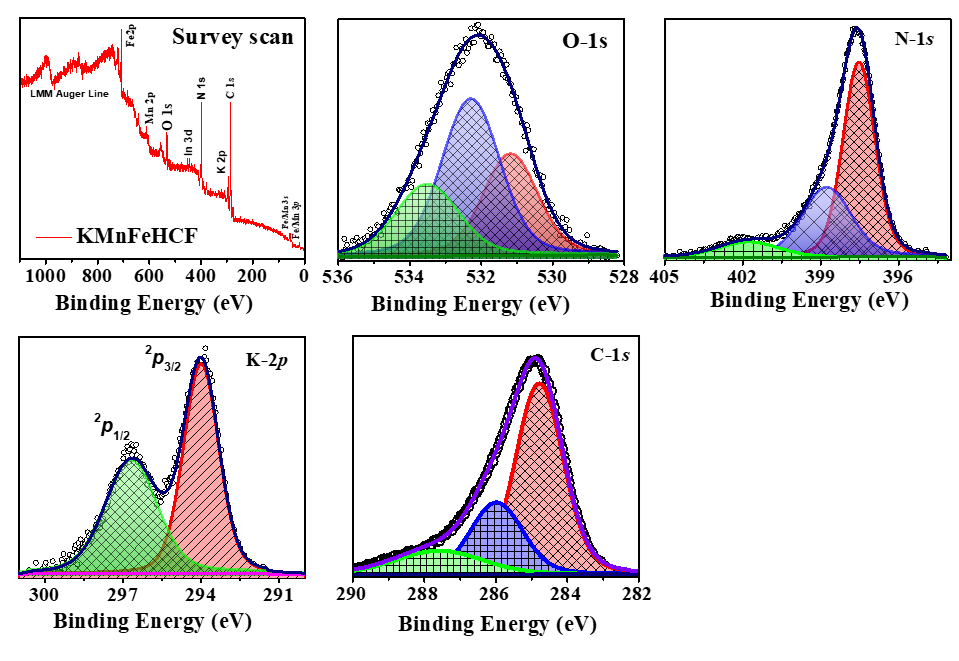


**Figure S14**: Survey scan and fitted core level XPS spectra of O-1s, N-1s and K-2p and C-1s of KMnFeHCF electrode after 100 GCD cycles.

**Figure S15**: Fitted room-temperature Mössbauer spectroscopy data recorded at different voltages of charging and discharging cycling of the KMnFeHCF electrode

**Table S2:** The comparison of the specific capacities of KMnFeHCF with respect to reported Prussian blue and its analogues in three-electrode/half-cell configuration for AAIBs.

| **Materials** | **Electrolyte** | **Oprating Window** | **Sp. Capacity**  **mAhg^-1^** | **Current Density** | **Capacity Retention** | **Reference** |
| --- | --- | --- | --- | --- | --- | --- |
| **KMnFeHCF** | **0.1 *M* (NH₄)₂SO₄** | **-0.2 to 1 V** | **145**  **130** | **3 A/g**  **5 A/g** | **97 % after 300 cycles** | **This work** |
| CuHCF | 2 M NH_4_OTf@S | -0.3 to 1.2 V | 81.5 | 0.1 A/g | 70.7 % after 500 cycles | [1] |
| NiHCF | 2 M Ammonium Acetate | 0 to 0.8 V | 61.3 | 50 mA/g | 71.0% after 1000 cycles | [2] |
| Cu_x_Ni_2–x_Fe(CN)_6_ | 1 M (NH_4_)_2_SO_4_ | 0.5 to 1.0V | 61.6 | 0.3 C | 81.8% after 120 cycles | [3] |
| MnPBA-TA | 1 M (NH_4_)_2_SO_4_  + 0.1 M MnSO_4_ | −0.1 to 0.9 V | 122 | 1 A/g | 80% after 200 cycles | [4] |
| CNCMF-PBAs | 5.8 M (NH_4_)_2_SO_4_ | 0 to 1.05 V | 101.2 | 20 C | 100% after 1000 cycles | [5] |
| Mn-PBA@ppy | 2.0 m NH_4_Otf | -0.2 to 1 V | 72 | 0.1A/g | 94% after 300 cycles | [6] |
| MnHCF | 1 M NH_4_TFSI | 0 to1.0 V | 104 | 0.1A/g | 98% after 100 cycles | [7] |
| N-FeMnHCF | 21 m NH_4_TFSI | 0 to1.2 V | 68 | 0.1A/g | ≈90% after 300 cycles | [8] |
| FeMnHCF | 24 M NH_4_CF_3_SO_3_ | −0.5–1.3 V | 123.8 | 0.5 A/g | 68.5% after 10 000 cycles | [9] |
| MnCu-PBA | 1 M NH_4_Cl | 0.1 to 1.1 V | 78 | 2 A/g | 100% after 10000 cycles | [10] |
| N-HEPBA | 1 M (NH_4_)_2_SO_4_  + 5 mM CuSO_4_ | 0 to1.2 V | 129 | 0.1 A/g | 100% after 1000 cycles | [11] |
| K-V-Fe PBA | 1 mol/L  (NH_4_)_2_SO_4_ | −0.5 to 1.2 V | 92.85 | 2 A/g | 91.44% 2,000 cycles | [12] |
| FeHCF | Fe_2_(SO_4_)_3_  + (NH_4_)_2_SO_4_ | 0 to 0.8 V | 80 | 50 mA/g | 96.3% retention after ~1,000 cycles | [13] |

**References : Table S2**

[1] **A Sustainable NH_4_^+^ Ion Battery by Electrolyte Engineering**, Z Tian, J Yin, T Guo, Z Zhao, Y Zhu, Y Wang, J Yin, Y Zou, Y Lei, J Ming, O Bakr, O F. Mohammed, H N. Alshareef, *Angewandte Chemie International Edition* 61, e202213757, 2022

[2] **Ligand Field-Induced Dual Active Sites Enhance Redox Potential of Nickel Hexacyanoferrate for Ammonium Ion Storage**, M Zhou, T Wu, M Kang, T Cheng, H Li, L He, C Lian, T Ma, Q Zhao, *Advanced Materials*, 37, 2419446, 2025.

[3] **Cu-substituted nickel hexacyanoferrate with tunable reaction potentials for superior ammonium ion storage,** L Fan, G Shu, Y Liu, H Yu, L Yan, L Zhang, J Shu, *Journal of Materials Science & Technology*, 169, 19-27, 2024.

[4] **Tailoring surface structures in Mn-based Prussian blue analogues for enhanced NH_4_+ transport and high-performance aqueous batteries,** J Yang, H Fu, L Ye, M Shi, E Huixiang Ang, *Mater. Horiz*.,12, 8565-8576, 2025.

[5] **Ultralong-Life Aqueous Ammonium-Ion Batteries Enabled by Unlocking Inert-Site of Medium-Entropy** **Prussian Blue Analogs**, C-Yan Wei, Z-Hui Sun, Z-Yi Gu, D-Xue Han, L Niu, X-Long Wu, *Advanced Energy Materials,* 15, Issue22, 2500589, 2025..

[6] **Encapsulation of Prussian Blue Analogues with Conductive Polymers for High-performance Ammonium-Ion Storage,** Q Liu, D Zhang, Y Yang, Y Gu, Z Liang, W Chen, Y Wu, L Hu, *Advanced Energy Materials* 15, 2402863, 2025.

[7] **Organic Ammonium Ion Battery: A New Strategy for a Nonmetallic Ion Energy Storage System** H. Zhang, Y. Tian, W. Wang, Z. Jian, W. *Chen, Angew. Chem., Int. Ed*., 61, 202204351, 2022.

[8] **How Prussian Blue Analogues Can Be Stable in Concentrated Aqueous Electrolytes,** L. Chen, W. Sun, K. Xu, Q. Dong, L. Zheng, J. Wang, D. Lu, Y. Shen, J. Zhang, F. Fu, *ACS Energy Lett*. 7, 1672, 2022

[9] **Coupling dual metal active sites and low-solvation architecture toward high-performance aqueous ammonium-ion batteries,** L. Du, S. Bi, M. Yang, Z. Tie, M. Zhang, Z. Niu, *Proc. Natl. Acad. Sci. USA*, 119, 2214545119, 2022.

[10] **Boosting Performance of Aqueous Ammonium Ion Batteries via Mn-Partial-Substituted Cu Prussian Blue Cathode,** Y Shi, H Qiu, Z Xu, M Chen, H Gao, X Yu, J Xu, J Cao, *ACS Sustainable Chem. Eng*., 13, 34, 14162–14169, 2025..

[11] **Unlocking Prussian Blue Analogues Inert-Site to Achieve High-Capacity Ammonium Storage,** Y Shen, J Zou, H Lan, Y Ding, Z Liang, Z Yang, Z Zeng, J Long, Y Zhao, L Fu, M Zeng, *Advanced Functional Materials* 34, 2400598, 2024.

[12] **Novel K-V-Fe Prussian blue analogues nanocubes for high-performance aqueous ammonium ion batteries,** Junjie Xing, Xiuli Fu, Shundong Guan, Yu Zhang, Ming Lei, Zhijian Peng, *Applied Surface Science* 543, 148843, 2021.

[13] **Common ion effect enhanced Prussian blue analogues for aqueous ammonium ion storage,**

S Li, M Xia, C Xiao, X Zhang, H Yu, L Zhang, Jie Shu, *Dalton Trans.,* 50, 6520-6527, 2021.

**Table S3:** The comparison of electrochemical performance for ammonium-ion full cells based on Prussian blue and its analogues.

| **Full cell** | **Capacity**  **(mAh/g)** | **Energy Density**  **(Wh/kg)** | **Power Density**  **(W/kg)** | **Capacity retention**  **Over cycles** | **Reference** |
| --- | --- | --- | --- | --- | --- |
| **KMnFeHCF//Graphite** | **71@1.25 A/g**  **51@2.2A/g** | **32.9** | **1103** | **50 % over 1850 cycles** | **This Work** |
| NiHCF//AC | 62.2@50 mA/g | 56 | - | 100% over 1400 cycles | [1] |
| MnCu-PBA//BPD | 61 @ 0.1A/g | 33.4 | - | 95.2% over 4000 Cycles | [2] |
| Mn–PBA–TA//PQANS | 42.6 @ 1 A/g | 36.2 | 849.8 | 88.5% over 1000 cycles | [3] |
| NiAPW((NH_4_)_1.47_Ni[Fe(CN)_6_]_0.88_)//PTCDI | 41 @ 0.06 A/g | 43 | - | 67 % over 1000 cycles | [4] |
| PTPD//CuFe-PBA | 206.6@0.5 A/g | 109 | 502.38 | 74.5% over 3000 cycles | [5] |
| CuHCF//PANI | 56.1 | - | - | 95.1% over 1240 cycles | [6] |
| CuHCF//PTCDI | 39 @ 0.3 A/g | 41.5 | - | 77% after 2000 cycles | [7] |
| FeMnHCF//PTCDI | 123.8 @0.5A/g | 71 | - | 47.7 mAh/g after 3,000 cycles at 9.0 A/g | [8] |
| CNCMF-PBAs//PTCDI | 65.7 @ 10 C | - | - | 85% after 4000 | [9] |
| N-CuMnHCF//PTCDI | 67.23 | 44.44 | 197.47 | 96.23 % over 10,000 cycles | [10] |
| A-PBA//PTCDI | 50.8@400 mA/g | 55.5 | 3600 | 72.3% after 4000 cycles | [11] |

**References: Table S3**

[1] **Ligand Field-Induced Dual Active Sites Enhance Redox Potential of Nickel Hexacyanoferrate for Ammonium Ion Storage**, M Zhou, T Wu, M Kang, T Cheng, H Li, L He, C Lian, T Ma, Q Zhao, *Advanced Materials*, 37, 2419446, 2025.

[2] **Boosting Performance of Aqueous Ammonium Ion Batteries via Mn-Partial-Substituted Cu Prussian Blue Cathode,** Y Shi, H Qiu, Z Xu, M Chen, H Gao, X Yu, J Xu, J Cao, *ACS Sustainable Chem. Eng*., 13, 34, 14162–14169, 2025.

[3] **Tailoring surface structures in Mn-based Prussian blue analogues for enhanced NH4+ transport and high-performance aqueous batteries,** J Yang, H Fu, L Ye, M Shi, E H Ang, *Mater. Horiz*., 2025.

[4] **Rocking-Chair Ammonium-Ion Battery: A Highly Reversible Aqueous Energy Storage System** X Wu, Y Qi, J J. Hong, Z Li, A S. Hernandez, X Ji, *Angewandte Chemie International Edition*, 56, Issue42, 13026-13030, 2017.

[5] **Conjugated Enhanced Polyimide Enables High-Capacity Ammonium Ion Storage,** F Huang, W Zhao, Y Guo, Y Mi, S Gull, G Long, P Du, *Advanced Functional Materials*, Volume34, Issue44, October 29, 2407313, 2024.

[6] **Hydrogen Bond-Assisted Ultra-Stable and Fast Aqueous NH4+ Storage**, X Zhang, M Xia, H Yu, J Zhang, Z Yang, L Zhang & J Shu, *Nano-Micro Latters* Volume 13, article number 139, 2021.

**[7] A Sustainable NH_4_^+^ Ion Battery by Electrolyte Engineering**, Z Tian, J Yin, T Guo, Z Zhao, Y Zhu, Y Wang, J Yin, Y Zou, Y Lei, J Ming, O Bakr, O F. Mohammed, H N. Alshareef, *Angewandte Chemie International Edition* 61, e202213757, 2022.

**[8] Coupling dual metal active sites and low-solvation architecture toward high-performance aqueous ammonium-ion batteries** L. Du, S. Bi, M. Yang, Z. Tie, M. Zhang, Z. Niu, *Proc. Natl. Acad. Sci. USA*, 119, 2214545119, 2022.

[9] **Ultralong-Life Aqueous Ammonium-Ion Batteries Enabled by Unlocking Inert-Site of Medium-Entropy** **Prussian Blue Analogs**, C-Yan Wei, Z-Hui Sun, Z-Yi Gu, D-Xue Han, L Niu, X-Long Wu, *Advanced Energy Materials,* 15, Issue22, 2500589, 2025.

**[10] Exposed crystal facet regulation of Prussian blue analogues by pre-intercalation NH4+ for high-performance aqueous ammonium-ion batteries,** B Tian, X Guan, L Yang, D Wang, J Zhang, J Zhang, X Wang, J Nie, P Yin, G Wang, *Chemical Engineering Journal*, 520, 165854, 2025.

**[11]** **How Prussian Blue Analogues Can Be Stable in Concentrated Aqueous Electrolytes** L. Chen, W. Sun, K. Xu, Q. Dong, L. Zheng, J. Wang, D. Lu, Y. Shen, J. Zhang, F. Fu, *ACS Energy Lett*., 7, 1672, 2022.


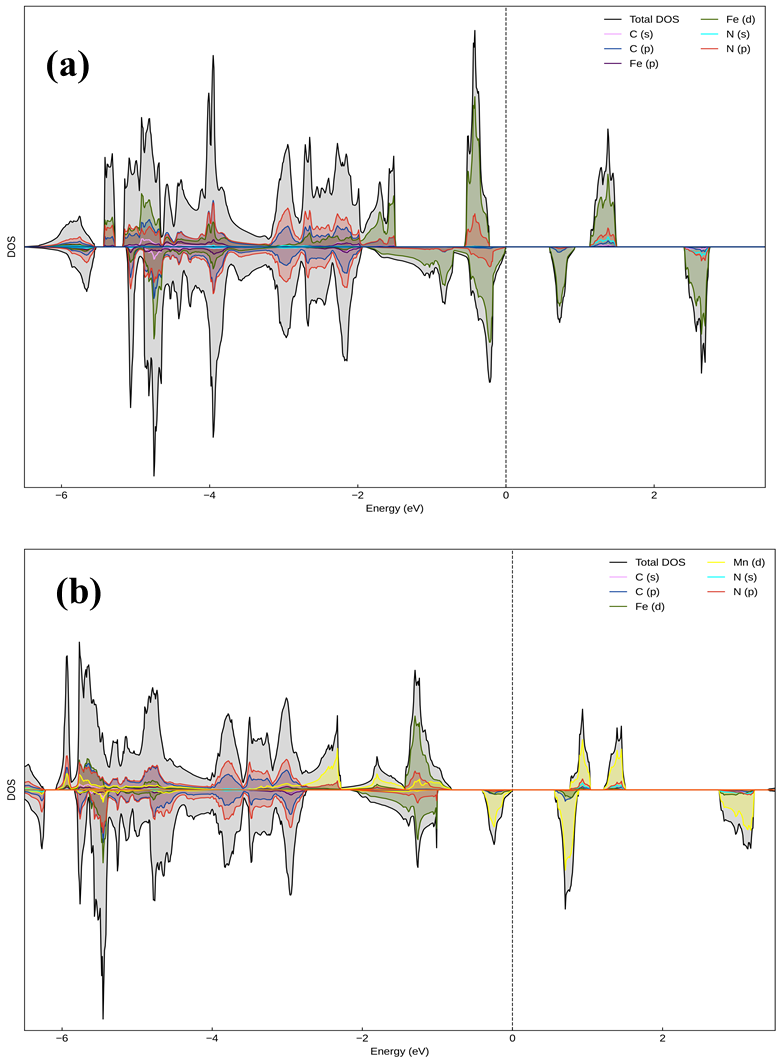


**Figure S16**: Total and partial DOS for the KFeHCF (a), and KMnHCF (b) compounds.
